# Rapamycin Improves the Response of Effector and Memory CD8^+^ T Cells Induced by Immunization With ASP2 of *Trypanosoma cruzi*

**DOI:** 10.3389/fcimb.2021.676183

**Published:** 2021-05-25

**Authors:** Barbara Ferri Moraschi, Isaú Henrique Noronha, Camila Pontes Ferreira, Leonardo M. Cariste, Caroline B. Monteiro, Priscila Denapoli, Talita Vrechi, Gustavo J. S. Pereira, Ricardo T. Gazzinelli, Joseli Lannes-Vieira, Maurício M. Rodrigues, Karina R. Bortoluci, José Ronnie C. Vasconcelos

**Affiliations:** ^1^ Molecular Immunology Laboratory, Center of Molecular and Cellular Therapy, Federal University of São Paulo (UNIFESP), São Paulo, Brazil; ^2^ Department of Microbiology, Immunology and Parasitology, Federal University of São Paulo (UNIFESP), São Paulo, Brazil; ^3^ Recombinant Vaccines Laboratory, Department of Biosciences, Federal University of São Paulo, Santos, Brazil; ^4^ Department of Pharmacology, Federal University of São Paulo, (UNIFESP), São Paulo, Brazil; ^5^ René Rachou Research Center, Fiocruz, Belo Horizonte, Brazil; ^6^ Division of Infectious Disease and Immunology, Department of Medicine, University of Massachusetts Medical School, Worcester, MA, United States; ^7^ Laboratoy of Biology of the Interactions, Oswaldo Cruz Institute, Fiocruz, Rio de Janeiro, Brazil

**Keywords:** rapamycin, mTOR, CD8^+^ T-cells, vaccine, *Trypanosoma cruzi*, effector CD8+ T cells, memory CD8+ T cells

## Abstract

Deficiency in memory formation and increased immunosenescence are pivotal features of *Trypanosoma cruzi* infection proposed to play a role in parasite persistence and disease development. The vaccination protocol that consists in a prime with plasmid DNA followed by the boost with a deficient recombinant human adenovirus type 5, both carrying the ASP2 gene of *T. cruzi*, is a powerful strategy to elicit effector memory CD8^+^ T-cells against this parasite. In virus infections, the inhibition of mTOR, a kinase involved in several biological processes, improves the response of memory CD8^+^ T-cells. Therefore, our aim was to assess the role of rapamycin, the pharmacological inhibitor of mTOR, in CD8^+^ T response against *T. cruzi* induced by heterologous prime-boost vaccine. For this purpose, C57BL/6 or A/Sn mice were immunized and daily treated with rapamycin for 34 days. CD8^+^ T-cells response was evaluated by immunophenotyping, intracellular staining, ELISpot assay and *in vivo* cytotoxicity. In comparison with vehicle-injection, rapamycin administration during immunization enhanced the frequency of ASP2-specific CD8^+^ T-cells and the percentage of the polyfunctional population, which degranulated (CD107a^+^) and secreted both interferon gamma (IFNγ) and tumor necrosis factor (TNF). The beneficial effects were long-lasting and could be detected 95 days after priming. Moreover, the effects were detected in mice immunized with ten-fold lower doses of plasmid/adenovirus. Additionally, the highly susceptible to *T. cruzi* infection A/Sn mice, when immunized with low vaccine doses, treated with rapamycin, and challenged with trypomastigote forms of the Y strain showed a survival rate of 100%, compared with 42% in vehicle-injected group. Trying to shed light on the biological mechanisms involved in these beneficial effects on CD8^+^ T-cells by mTOR inhibition after immunization, we showed that *in vivo* proliferation was higher after rapamycin treatment compared with vehicle-injected group. Taken together, our data provide a new approach to vaccine development against intracellular parasites, placing the mTOR inhibitor rapamycin as an adjuvant to improve effective CD8^+^ T-cell response.

## Introduction

The immunization regimen known as heterologous prime-boost vaccination uses two distinct vectors for priming and boosting, both carrying the target antigen. Different combinations of vectors have been tested and the application of this strategy has promoted an immune response against several experimental infections, such as simian immunodeficiency virus (SIV), malaria, Ebola, tuberculosis, Chagas disease, toxoplasmosis and COVID-19 (Li et al., 1993; McConkey et al., 2003; Wilson et al., 2006; Zhang et al., 2007; De Alencar et al., 2009; Elvang et al., 2009; Hensley et al., 2010; Hill et al., 2010; Martins et al., 2010; Chuang et al., 2013; Graham et al., 2020). This regimen began to be studied more than 20 years ago and has shown excellent protective responses both to intracellular pathogens and neoplastic cells due to the induction of cytotoxic CD8^+^ T-cells (Zavala et al., 2001; Gilbert et al., 2002; Ranasinghe and Ramshaw, 2009).

Chagas disease, caused by the intracellular protozoan *Trypanosoma cruzi*, is an endemic disease in Latin America and considered a neglected one by the World Health Organization (WHO), as it affects approximately 6-7 million people worldwide (World Health Organization, 2016). The clinical course of Chagas disease generally comprises acute and chronic phases and affects mainly the heart and the digestive system. Currently, the treatment consists of administering the chemotherapeutic benznidazole or nifurtimox, but these drugs have limited efficacy when started late, and there are still no vaccines for the disease (Pérez-Molina and Molina, 2017).

The heterologous prime-boost vaccination protocol, capable of conferring a significant degree of protection against experimental *T. cruzi* infection, consists of priming immunization with plasmid DNA, followed by boosting with replication-defective human recombinant adenovirus type 5, both vectors expressing the amastigote surface protein-2 (ASP2) (De Alencar et al., 2009; Haolla et al., 2009; Dominguez et al., 2011; Rigato et al., 2011; Vasconcelos et al., 2012; Ferreira et al., 2017). Previously, we demonstrated that this prime-boost protocol generates a high frequency of effector CD8^+^ T cells (CD44^High^, CD11a^High^, CD62L^Low^, CD127^Low^ and KLRG1^High^), which subsequently acquire an effector memory phenotype (CD44^High^, CD11a^High^, CD62L^Low^, CD127^+^ and KLRG1^High^) (Rigato et al., 2011). These phenotype and cytotoxic effector activity were long-lasting, being detected 98 days after boosting (De Alencar et al., 2009; Rigato et al., 2011). Moreover, the effector memory CD8^+^ T-cells (TEM) induced by heterologous prime-boost immunization are polyfunctional, since express IFNγ , TNF and CD107a, and play cytotoxic activity simultaneously (De Alencar et al., 2009; Rigato et al., 2011).

During the specific immune response development, several signaling pathways are required to activate T-cells and initiate their differentiation. The highly conserved kinase called mammalian target of rapamycin (mTOR) is a key regulator of essential cellular processes such as cell growth, autophagy, survival, proliferation, and metabolism in response to environmental factors, including levels of cellular energy, insulin, cytokines and amino acids, through the complexes mTORC1 and mTORC2, that contain different scaffold associated proteins, Raptor and Rictor, respectively, which define their downstream targets pathways (Dennis et al., 2001; Wullschleger et al., 2006; Thomson et al., 2009; Powell et al., 2012). In CD8^+^ T-cells, mTORC1 controls, for example, glucose uptake and glycolysis during activation and effector phases and also participates in the signaling generated by the antigen recognition receptor (TCR) and cytokines (Jacobs et al., 2008; Buck et al., 2015; Chang and Pearce, 2016). Even though rapamycin has been commonly used in organ transplantation to prevent graft rejection (Augustine et al., 2007), several studies have reported that mTOR inhibition by treatment with low and continuous doses of rapamycin during the immune challenge of CD8^+^ T cells could improve the function and memory formation following viral infections or tumor challenges (Araki et al., 2009; Rao et al., 2010; Li et al., 2011; Turner et al., 2011; Bassett et al., 2012; Mattson et al., 2014; Shrestha et al., 2014).

The transcriptome of activated CD8^+^ T cells treated with rapamycin revealed that most genes modulated by mTOR inhibitor are associated with apoptosis, survival, maintenance, and cell migration, which take part in the CD8^+^ T cell programming after activation (Mattson et al., 2014; Borsa et al., 2019). Moreover, a clinical trial found that elderly people immunized against influenza and treated with rapamycin had a response 20% higher in antibody titer than the placebo group, paralleled by increased T cells life span, improving immune function and reducing infections (Mannick et al., 2014; Mannick et al., 2018).

Based on these findings, here we tested the role of the mTOR inhibitor rapamycin combined with heterologous prime-boost immunization against *T. cruzi* during CD8^+^ T-cell activation. For this purpose, C57BL/6 and AS/n mice were immunized and daily treated with rapamycin. The inflammatory and cytolytic immune responses were analyzed. Additionally, mice were challenged with the highly infective trypomastigote forms of Y strain.

## Methods

### Ethics Statement

This study was carried out in strict accordance with the recommendations in the Guide for the Care and Use of Laboratory mice of the Brazilian National Council of Animal Experimentation (http://www.sbcal.org.br/) and Federal Law 11.794 (October 8, 2008). The project was approved by the Ethical Committee for Animal Experimentation at the Federal University of Sao Paulo, registered under number 9959021014.

### Mice and Parasites

Male and female 8-week-old C57BL/6 and A/Sn mice were supplied by the Center for the Development of Experimental Models for Medicine and Biology (CEDEME) from the Federal University of São Paulo. Blood trypomastigotes of Y type II strain of *T. cruzi* were maintained by weekly passages in A/Sn mice at the Xenodiagnostic Laboratory of Dante Pazzanese Institute of Cardiology. For *in vivo* experiments, the challenge was performed with 150 or 1 x 10^4^ trypomastigotes diluted in PBS (0.2 mL) in A/Sn and C57BL/6 mice, respectively, administered subcutaneously (s.c.) in the tail. Parasitemia was monitored after the 6^th^ day of infection until day 15. A blood sample (5 μl) was collected from the tail for parasite quantification on the light microscope.

### Immunization Protocol

The heterologous prime-boost immunization protocol previously described by Rigato and group (Rigato et al., 2011) was used in this study. The protocol consists of a dose of plasmid DNA, with the vectors pcDNA3 (control) or pIgSPClone9, at 10 or 100 μg/mouse. Three weeks after the first immunization, mice were immunized with 2 x 10^7^ or 2 x 10^8^ pfu of the adenoviral vectors Adβ-Gal (control) or AdASP-2. Both immunizations were performed by intramuscular route in the Tibialis anterior muscle. Experimental groups were delineated as follows: 1) Control: immunized with the control vectors pcDNA3 and Adβ-Gal; 2) ASP2: immunized with pIgSPCl.9/AdASP-2 and vehicle-injected (PBS); 3) ASP2/rapamycin: immunized with pIgSPCl.9/AdASP-2 and rapamycin-treated.

### Treatment With Rapamycin

Mice were treated every 24 hours with 2 μg rapamycin (Sigma Aldrich) per mouse (0.075 mg/kg/day), diluted in 0.2 mL PBS *via* intraperitoneal (i.p.) for 34 days, starting at priming (Li et al., 2011; Bassett et al., 2012). Control mice were treated with the vehicle (PBS). To assess mTOR inhibition, phospho-S6 ribosomal protein conjugate antibody (Ser235/236) from Cell Signaling Technology was used. This antibody binds to the PS6 protein only in its phosphorylated form, indicating whether mTOR was activated. For phospho-S6 ribosomal protein staining, the protocol was performed according to Ersching and group (Ersching et al., 2017).

### Peptides and Multimers

The ASP2 synthetic peptides VNHRFTLV and TEWETGQI were synthesized by GenScript with purity greater than 95%. The peptides were used during *in vivo* and *ex vivo* assays to stimulate specific CD8^+^ T-cells.

H2K^b^-VNHRFTLV multimer was purchased from ProImmune Inc., and H2K^K^-TEWETGQI multimer, labeled with allophycocyanin, was purchased from Immudex. Both were used for labeling TCR-specific CD8^+^ T-cells.

### Flow Cytometry Analysis

Splenocytes were treated with ACK buffer (NH_4_Cl, 0.15 M, KHCO_3_, 10 mM, 0.1 mM Na2-EDTA, pH 7.4) for osmotic lysis of red cells and washed with RPMI supplemented with 10% fetal bovine serum (FBS). After lysis, cells were labeled with the multimers for 10 minutes at room temperature. The cell surface was stained for 30 min at 4°C with the following antibodies: anti-CD8 (clone 53-67); anti-CD11a (clone 2D7), anti-CD11c (clone HL3), anti-CD25 (clone 7D4), anti-CD27 (clone LG.7F9), anti-CD31 (clone MEC13.3), anti-CD43 (clone Ly 48), anti-CD43 (clone 1B11), anti-CD44 (clone IM7), anti-CD49d (clone R-12), anti-CD62L (clone MEL-CD70 (clone FR70), anti-CD95 (clone Jo2), anti-CD95L (MFL3), anti-CD122 (clone TM-b1), anti-CD127 (Clone J43), anti-PDL-1 (clone MIH5), anti-CCR-5 (clone HM-CCR5), anti-CCR-7 (clone 4B12), anti-KLRG-1 (clone 2F1 and anti-CD183 (CXCR3-clone 173). At least 500,000 events were acquired on FACS Canto II flow cytometer (BD). The results were analyzed with FlowJo software version 9.9.6 (FlowJo, LLC).

### Intracellular Cytokine Staining

Two million splenocytes were incubated in the presence or absence of the peptides VNHRFTLV or TEWETGQI (10 μg/mL or 10 μM) in supplemented RPMI medium with CD107a FITC antibody (clone 1D4B, BD), anti-CD28 (clone 37.51, BD Bioscience), BD Golgi-Plug (1 µL/mL, BD Bioscience) and monensin (5 µg/mL, Sigma Aldrich) no longer than 12 hours in V-bottom 96-well plates (Corning) in a final volume of 200 µL, at 37°C containing 5% CO_2_. After incubation, cells were labeled with anti-CD8 antibody PerCP (clone 53-6.7, BD) for 30 minutes at 4°C. For cellular fixation and permeabilization, the Cytofix/Cytoperm kit (BD Biosciences) was used according to the supplier’s instructions. For intracellular staining, was used the following antibodies: anti-IFNγ APC (clone XMG1.2, BD Biosciences) and anti-TNF PE (clone MPC-XT22, BD Biosciences). At least 700,000 events were acquired using a FACSCanto II flow cytometer (BD Biosciences).

### Enzyme-Linked-Immunospot Assay (ELISpot)

The IFNγ secretion was measured by ELISpot as described previously (Ferreira et al., 2017). Briefly, 10^5^ responder cells (represented by splenocytes from mice previously immunized) were incubated with 3 x 10^5^ antigen-presenting cells (represented by splenocytes from naive mice) on nitrocellulose 96-wells flat-bottom plates (Millipore) in the presence or absence of the specific ASP2 peptides VNHRFTLV or TEWETGQI for CD8^+^ T cells for 24 hours. The number of IFNγ-producing cells was determined using a stereoscope. The final value refers to the numeric average of spots of stimulated wells minus the numeric average of spots of unstimulated wells. The result is multiplied by 10 to display the data by spot forming cells (SFC) in million units.

### Cytokine Determination

One million splenocytes were incubated for 48 hours in the presence or absence of the peptide VNHRFTLV in a final concentration of 10 μg/mL. Culture supernatants were harvested and stored at −80°C until analysis. IL-2, IL-4, IL-6, IL-10, IL-17, IFNγ, and TNF cytokines were detected simultaneously using mouse Th1/Th2/Th17 cytokine bead array (CBA) kit (BD Pharmingen), according to the manufacturer’s instructions. After acquiring samples on a flow cytometer, the data were analyzed in FCAP Array™ software to generate results in graphical and tabular format. The data are expressed in pg/mL and the values correspond to the number of the value of stimulated samples minus the value of unstimulated ones.

### *In Vivo* Cytotoxicity Assay

Splenocytes from naive mice were divided into two populations stained with carboxyfluorescein succinimidyl ester (CFSE - Molecular Probes) at a final concentration of 10 μM (CFSE^High^) and 1 μM (CFSE^Low^). PKH26 Red Fluorescent Cell Linker (Sigma-Aldrich) was also used at a final concentration of 20 μM. The target cells labeled with CFSE^High^ or PKH were pulsed with peptide VNHRFTLV or TEWETGQI for 40 minutes at 37°C according to each experiment’s concentration. CFSE^Low^ cells remained uncoated. Subsequently, all stained populations were counted and mixed at the same proportion. 4 x 10^7^ cells were transferred *via* intravenous into mice and after 14 hours, the spleens of recipient mice were collected to CFSE^Low^, CFSE^High^ and PKH^+^ detection by flow cytometry using FACS Canto II. The percentage of specific lysis was determined by this formula:

$$\%Lysis=[1-(\%CFSE^{high}immunized/\%CFSE^{low}immunized)/(\%CFSE^{high}naive/\%CFSE^{low}naive)]\times 100$$

### BrdU Proliferation Assay

For *in vivo* proliferation, A/Sn mice were treated with 2 mg of BrdU (SIGMA) (i.p.) after boosting for 14 days every 48 hours. Then, the splenocytes were purified for staining with anti-CD8 and BrdU detection according to the manufacturer’s instructions (BrdU Flow Kit APC or FITC - BD Pharmingen). At least 100,000 cells were obtained in low flow rate on a BD FACSCanto II flow cytometer (BD Bioscience), and then analyzed with FlowJo software (FlowJo, LLC).

### BMDC Generation, Immunophenotyping Antigen-Presentation Capacity

Bone marrow dendritic cells (BMDC) were generated as described earlier (Ersching et al., 2016). Overall, cells removed from the femurs were cultured with 20 ng/ml of recombinant granulocyte–macrophage colony-stimulating factor (rGM-CSF- RD System) for 7 days. The medium was replaced on the fourth day. In some conditions, BMDC were matured with 200 ng/ml of LPS from *E. coli* (Sigma-Aldrich) for 3 hours, resulting in a population that comprises around 80% of CD11c^+^ cells. For *in vitro* antigen presentation capacity assay, BMDC were incubated with AdASP-2 (MOI = 50) for 24 hours, or with VNHRFTLV peptide (10 μg/mL) for 1 hour. Rapamycin was used in a final concentration of 1 μM. The frequency of IFNγ-producing by CD8^+^ T cells was detected by ELISpot. Additionally, BMDC were stained with the following antibodies for flow cytometry analysis: anti-CD11c, anti-CD40, anti-CD86 and anti-MHC class I (H2K^b^).

### Statistical Analysis

Groups were compared using One-way ANOVA followed by Tukey’s HSD test on Vassarstats (http://vassarstats.net). Before performing parametric tests, the normal distribution was analyzed by Shapiro-Wilk test and residuals distribution in QQ-plots on GraphPad Prism 8.0. Survival analysis by Log-rank was also performed on GraphPad Prism 8.0. Because the variances were similar, values were expressed as mean ± standard deviations (SD). The expression of the receptors was compared by MFI (mean fluorescence intensity), and the MFI of naive group was taken as a baseline. Differences were considered significant when the *p* value was <0.05.

## Results

### Rapamycin Treatment Enhances the Number and Quality of Specific CD8^+^ T-Cells

Initially, the blockade of mTOR by rapamycin was confirmed by ribosomal protein S6 staining, which is a target of mTOR kinase. The detection antibody used binds to S6 ribosomal protein only in its phosphorylated form (Ser235/236), indicating whether there was mTOR activity. CD8^+^ T cells from splenocytes of C57BL/6 mice incubated with rapamycin for 1 hour showed a lower expression of phosphorylated S6K than untreated or concavalin A stimulated cells (**Supplementary Figure 1**).

T lymphocytes perform a strong antiparasitic role mediated by the secretion of IFNγ and other mediators that also participate in the parasite dissemination control. The CD8^+^ T-cells induced by heterologous prime-boost regimen are polyfunctional, as they exhibit cytotoxic activity and secrete the cytokines IFNγ and TNF simultaneously (De Alencar et al., 2009; Rigato et al., 2011). Based on these findings, we evaluated the production of cytokines IFNγ and TNF, as well as the degranulation by the expression of the CD107a molecule (LAMP-1) in VNHRFTLV peptide-specific CD8^+^ T-cells obtained from splenocytes of immunized mice. Experimental groups were delineated as follows: 1) Control: immunized with the control vectors pcDNA3 and Adβ-Gal; 2) ASP2: immunized with pIgSPCl.9/AdASP-2 and vehicle-injected (PBS); 3) ASP2/rapamycin: immunized with pIgSPCl.9/AdASP-2 and rapamycin-treated.

**Figure 1:**
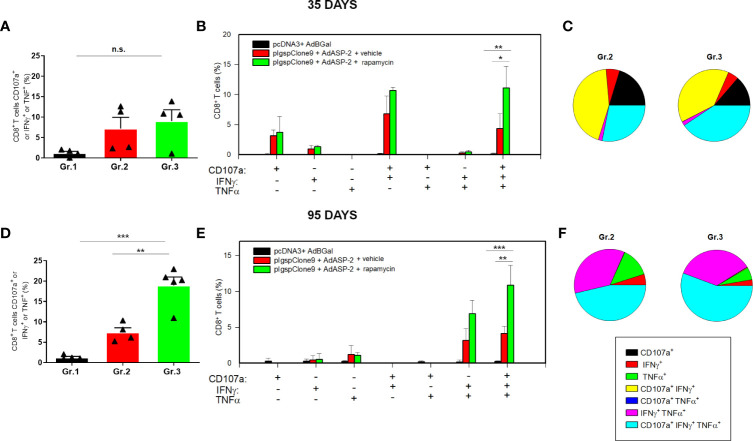
Specific CD8^+^ T cell-mediated immune responses was higher in rapamycin-treated mice, after 35 or 95 days from priming. C57BL/6 mice were immunized *via* i.m. with plasmid (100 µg) and adenovirus (2 x 10^8^ pfu) according to the experimental groups described in the *Methods* section. They were also treated daily with rapamycin or vehicle (i.p.) for 34 days. On days 35 or 95 after priming, splenic cells were collected and cultured *ex vivo* in the presence of anti-CD107a and anti-CD28, with or without the VNHRFTLV peptide. After 12 hours, the cells were labeled with anti-CD8, anti-IFNγ and anti-TNF antibodies. **(A, D)** Frequencies of CD8^+^ T cell that express CD107a or IFNγ or TNF after stimulation. **(B, E)** Subpopulations of CD8^+^ T cells expressing each individual molecule or the combinations between CD107a, IFNγ and TNF. **(C, F)** Pie charts show the fraction of specific cells expressing the indicated molecules. The results correspond to the mean values of 4 mice per group, with standard deviation. Statistical analysis was performed using the *One-Way* ANOVA and Tukey’s HSD tests. Asterisks indicate significant differences among groups, defined as *P<0.05, **P < 0.01, and ***P < 0.001. The experiments were repeated four times, and representative results are shown. N.S, non-significant. Boolean analysis was performed using FlowJo Software.

After *ex vivo* stimulation with the peptide VNHRFTLV and intracellular staining (gates strategy showed in **Supplementary Figure 2**), the number of CD8^+^ T-cells that simultaneously express IFNγ, TNF and CD107a, named polyfunctional subpopulation, were increased in rapamycin-treated mice (Gr.3), compared with vehicle-injected mice (Gr.2), after Boolean analysis (**Figures 1B, C**). In addition, the magnitude of responding CD8^+^ T-cells (frequency of cells that express at least one of the three molecules IFNγ or TNF or CD107a after *ex vivo* stimulus with the specific peptide) was also higher in Gr.3 (**Figure 1A**), at 35 days after priming. The differences between Gr.2 (vehicle-injected) and Gr.3 (rapamycin-treated) were more evident at 95 days after priming (**Figures 1D–F**).

**Figure 2:**
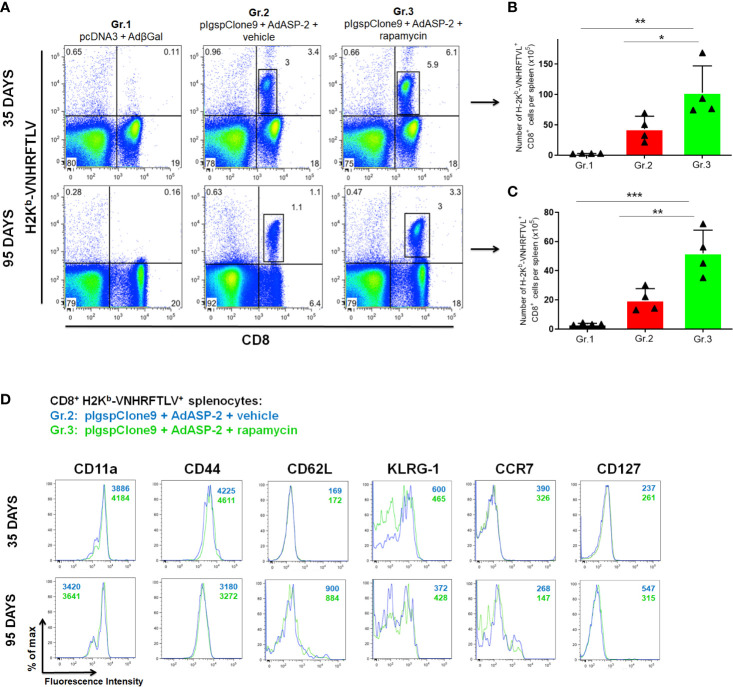
Frequency and absolute number of specific CD8^+^ T cells were higher in rapamycin-treated group, after 35 or 95 days from priming. C57BL/6 mice were immunized *via* i.m. with plasmid (100 µg) and adenovirus (2 x 10^8^ pfu) according to the experimental groups described in the method section. They were also treated daily with rapamycin or vehicle (i.p.) for 34 days. After 35 or 95 days of priming, the splenic cells were labeled with anti-CD8 and H2K^b^-VNHRFTLV pentamer for flow cytometry analysis. **(A)** Dot-plots charts correspond to the representative mouse (median) from 4 mice. Numbers represent frequencies of splenic cells. **(B, C)** The total number of specific lymphocytes was estimated. The bars indicate the group mean ± SD. Statistical analysis was performed using the One-Way ANOVA and Tukey’s HSD tests. Asterisks indicate significant differences among groups, defined as *P < 0.05, **P < 0.01, and ***P < 0.001. Data referring to 4 mice per group and representing 4 independent experiments. **(D)** Splenic cells were labeled with anti-CD8, H2K^b^-VNHRFTLV multimer and the molecules indicated above for flow cytometric analysis. Histograms show the expression of the markers in CD8^+^ H2K^b^-VNHRFTLV^+^ cells in Gr.2 (blue lines) and Gr.3 (green lines). Analyses were performed using cells pools of 4 mice and they are representative of two independent experiments. The numbers indicate the mean fluorescence intensity (MFI). The individual analysis of mice from each group presented similar results.

In order to measure the frequency of ASP2 specific CD8^+^ T-cells, the splenocytes were labeled with the H2K^b^-VNHRFTLV pentamer and anti-CD8. We found that the frequency and absolute number of specific CD8^+^ T-cells were significantly higher in Gr.3 after 35 days and sustained after 95 days after priming (**Figures 2A–C**). These results demonstrate that treatment with rapamycin enhances the response generated by immunization and memory formation, confirming that inhibition of mTOR modulates T-lymphocyte differentiation during adaptive immunity to *T. cruzi* antigens, as previously proposed in other conditions (Araki et al., 2009; Nam, 2009).

### Specific CD8^+^ T-Cells Phenotype Remains Unchanged After Treatment With Rapamycin

Traditionally, antigen-specific CD8^+^ T-cells are divided into three major groups according to their markers of activation, homing, migration, proliferation capacity and effector functions: i) effectors (TE): effector cells that control the infection (CD44^High^, CD11a^High^, CD62L^Low^, CD127^-^, KLRG1^High^); ii) central memory (TCM): long-lasting memory cells with high proliferative potential after antigen stimulation and reside in secondary lymphoid organs (CD44^High^, CD11a^High^, CD62L^High^,CD127^+^, KLRG1^High^); iii) effector memory (TEM): transitional cells that exist for a shorter time, present high effector activity and express TE surface markers (CD44^High^, CD11a^High^, CD62L^Low^, KLRG1^High^) and a TCM marker (CD127^+^). They home primarily in peripheral tissues and rapidly produce effector cytokines upon antigenic stimulation (Wherry et al., 2003; Lanzavecchia and Sallusto, 2005; Wirth et al., 2009; Wirth et al., 2010; Angelosanto and Wherry, 2010; Cui and Kaech, 2010; Ahmed and Akondy, 2011; Sheridan and Lefrançois, 2011).

Previously, it has been shown that heterologous prime-boost regimen induces a strong response of effector CD8^+^ T-cells, which develop into an effector memory (TEM) population (Rigato et al., 2011). Here, we challenged the hypothesis that mTOR inhibition changes the profile of CD8^+^ T-cells generated by vaccination as, for example, into a TCM phenotype, as described in other experimental models combined with rapamycin (Araki et al., 2009; Pearce et al., 2009; Turner et al., 2011). To this end, we performed the phenotypic characterization of specific CD8^+^ T-cells from the spleen of C57BL/6 mice immunized and treated with rapamycin or vehicle.

Specific CD8^+^ T-cells were stained with H2K^b^-VNHRFTLV multimer and anti-CD8. The expression pattern of the molecules CD11a, CD44, CD62L, CD127, KLRG-1 and CCR7 on specific CD8^+^ T-cells was similar between Gr.2 and Gr.3, at 35 and 95 days after priming (**Figure 2D**), showing that rapamycin, all in all, did not modify the TE(M) profile of CD8^+^ T-cells generated by vaccination. In both ASP2 immunized groups, differently from the expected, an increase in CD127 expression on specific memory CD8^+^ T cells was not found after 95 days. Other markers involved with activation and migration were not altered either. The complete immunophenotyping, where twenty-four surface markers associated with activation, regulation, migration, and cell death were used, is shown in **Supplementary Figures 3** and **4**.

### Cellular Response Remained High Even With Reduced Doses of Immunization in Rapamycin-Treated C57BL/6 Mice

**Figure 3:**
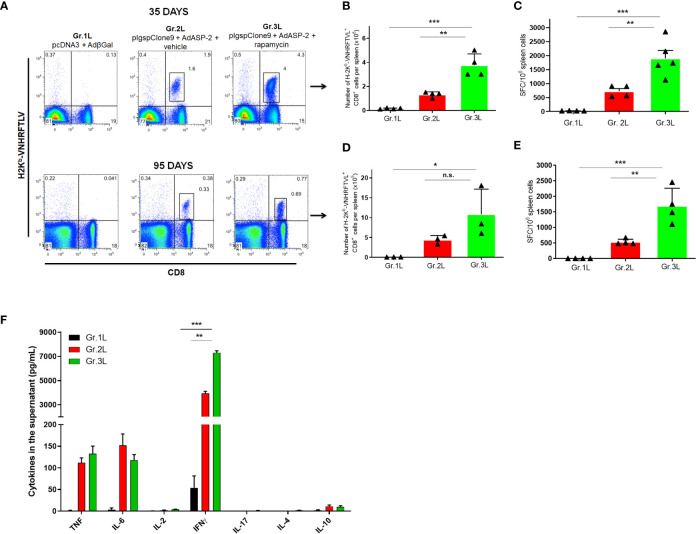
Rapamycin increased the frequency of specific CD8^+^ T cells and IFNγ production from mice immunized with reduced doses, after 35 or 95 days from priming. C57BL/6 mice were immunized *via* i.m. with plasmid (10 µg) and adenovirus (2 x 10^7^ pfu) according to the experimental groups described in the method section. They were also treated daily with rapamycin or vehicle (i.p.) for 34 days. On days 35 or 95 after priming, splenic cells were collected for surface staining of anti-CD8 and H2K^b^-VNHRFTLV^+^ pentamer or cultured with the specific peptide VNHRFTLV for ELISpot or CBA assay. **(A)** FACS charts show the frequency of CD8^+^ and H2K^b^-VNHRFTLV^+^ cells in the spleen. The dot-plots correspond to the representative mice (median) of the group. Numbers represent the frequencies of CD8^+^ H2K^b^-VNHRFTLV^+^ cells in the spleen. **(B, D)** The total number of specific CD8^+^ T cells was estimated. **(C, E)** Number of IFNγ producing cells by ELISpot. SFC: Spot-forming cells. **(F)** The supernatant of splenocytes from mice immunized for 95 days cultured for 48 hours was used to measure the indicated cytokines by flow cytometry. Statistical analysis was performed using the *One-Way* ANOVA and Tukey’s HSD tests. Asterisks indicate significant differences among groups, defined as *P < 0.05, **P < 0.01, and ***P < 0.001. Data referring to 4 mice per group and representing 4 independent experiments. N.S, Non-significant.

Next, we evaluated the role of rapamycin during vaccination with reduced doses, to highlight the adjuvant effect of rapamycin. For that purpose, C57BL/6 mice were vaccinated with 10-fold lower doses of plasmid/adenovirus, treated with rapamycin or vehicle and the immune response was assessed by the standard protocol. On days 35 and 95, splenocytes were isolated, and specific CD8^+^ T-cells were stained using H-2K^b^-restricted VNHRFTLV multimer. Splenocytes were stimulated with VNHRFTLV specific ASP-2 peptide for ELISpot anti-IFNγ. The increase in the number of VNHRFTLV-specific CD8^+^ T cells was replicated with 10-fold lower doses since the rapamycin-treated mice showed a higher frequency of specific CD8^+^ T cells (Gr.3L), both on days 35 and 95 after priming (**Figures 3A, B**). Later, when we analyzed the IFNγ secretion by CD8^+^ T-cells after *ex vivo* stimulation with the VNHRFTLV peptide, rapamycin-treated group (Gr.3L) was superior in both analyses (**Figures 3C–E**).

In addition, we determined the concentration of cytokines in the supernatant of VNHRFTLV-stimulated splenocytes from mice immunized 95 days after priming using Th1/Th2/Th17 CBA kit. IFNγ was the predominant cytokine after VNHRFTLV stimulation and the splenocytes from rapamycin-treated mice (Gr.3L) presented the highest IFNγ concentration in the supernatants (**Figure 3F**). As previously shown, heterologous prime-boost vaccination induces predominantly a Th1 profile response (De Alencar et al., 2009). Thus, the present findings enable us to adopt a protocol with reduced doses of vaccine formulation, which were able to induce a strong and potentially CD8^+^ T response.

### Higher *In Vivo* Cytotoxicity of Specific CD8^+^ T After the Rapamycin Treatment

Another essential effector function involved in intracellular pathogens dissemination control is the direct cytotoxicity performed by NK and CD8^+^ T cells, releasing cytotoxic granules, such as perforins, granzymes B and granulysin, which are responsible for forming pores on the plasma membrane of target infected cells as well as inducing apoptosis. Thus, the cytotoxic activity of VNHRFTLV-specific CD8^+^ T-cells in C57BL/6 mice immunized with 10-fold lower doses of plasmid/adenovirus and treated with rapamycin or vehicle was analyzed at 35 days after priming. The *in vivo* cytotoxicity assay was evaluated using adoptive transfer of labeled cells with two concentrations of CFSE dye, CFSE^High^ and CFSE^Low^. Only the CFSE^High^ population was pulsed with 2,5 μM of the VNHRFTLV ASP2 peptide. Both populations were transferred to recipient mice of the experimental groups (Gr.1L, Gr.2L and Gr.3L) and, after 15 hours, we analyzed the percentage of lysis in CFSE^High^ cells. The cytotoxic activity of VNHRFTLV-specific CD8^+^ T-cells was similar in both immunized groups, e.g., 97,6% in Gr.2L and 95,07% in Gr.3L (**Supplementary Figure 5**), which corroborates with our previous findings showing a strong cytotoxic activity induced by heterologous prime-boost immunization using the ASP2 (De Alencar et al., 2009).

**Figure 4:**
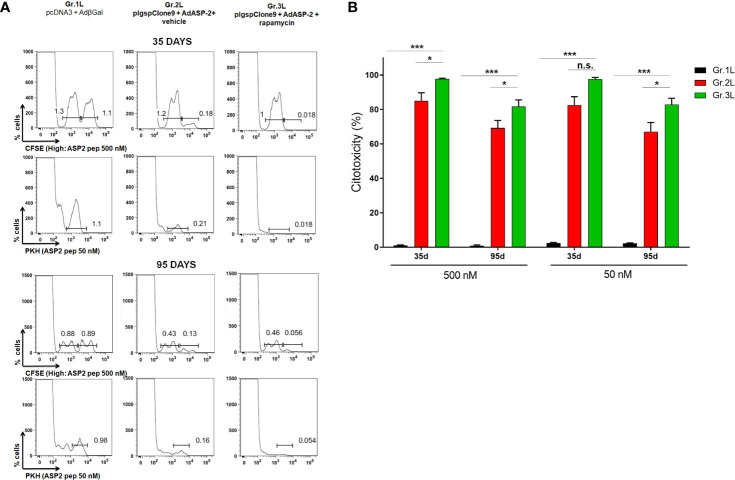
*In vivo* cytotoxicity of specific CD8^+^ T cells was higher in rapamycin-treated mice. C57BL/6 mice were immunized *via* i.m. with plasmid (10 µg) and adenovirus (2 x 10^7^ pfu) according to the experimental groups described in the method section. They were also treated daily with rapamycin or vehicle (i.p.) for 34 days. Splenocytes from naive mice were stained with CFSE or PKH. CFSE^High^ and PKH^+^ populations were pulsed with peptide VNHRFTLV at a final concentration of 500 nM or 50 nM, respectively. CFSE^Low^ was the negative control. Stained cells were transferred to the experimental groups and, after 14 hours, spleens were harvested to quantify the frequency of stained cells. Histograms represent the frequencies of CFSE^High^, CFSE^Low^ and PKH^+^ cells in each group, after 35 days **(A)** or 95 days from priming **(B)**. Percentage of cytotoxicity, with mean ± SD. Statistical analysis was performed using the One-Way ANOVA and Tukey’s HSD tests. Asterisks indicate significant differences among groups, defined as *P < 0.05, and ***P < 0.001. Results from 2 experiment and 4 mice per group. N.S, Non-significant.

Therefore, we performed another cytotoxicity assay lowering the concentrations of VNHRFTLV peptide, trying to reveal any beneficial effect of rapamycin treatment on cytotoxic activity. For that, two dyes were used to sort 3 different populations: CFSE^Low^, CFSE^High^ and PKH^+^. Both CFSE^High^ and PKH^+^ populations were pulsed with the peptide at a final concentration of 500 nM and 50 nM, respectively (**Figure 4**). After 15 hours, rapamycin-treated mice (Gr.3L) exhibited a higher percentage of cytotoxic activity against VNHRFTLV^+^ target cells of CFSE^High^ (500 nM peptide) and PKH^+^ (50 nM peptide) populations compared with the cytotoxic activity observed in vector control Gr.1L and vehicle-injected Gr.2L mice (**Figures 4A, B**). Similar results were found at 95 days after priming. As expected, the main difference between the two immunized groups was the cytotoxic activity of CD8^+^ T-cells on the PKH^+^ target cell population, pulsed with the lowest concentration (50 nM) of the specific VNHRFTLV peptide (**Figure 4B**).

### Treatment With Rapamycin Increased the Survival of the Highly Susceptible A/Sn Mice Immunized With Low Doses After Challenge

Once the putative protective profiles of CD8^+^ T-cells induced by vaccination have been improved when combined with rapamycin treatment, we challenged highly susceptible A/Sn mice with *T. cruzi* and survival was registered. Since heterologous prime-boost immunization can induce a protective response, mice groups were immunized with 10-fold lower doses of plasmid/adenovirus and treated with rapamycin or vehicle. Thirty-five days after priming, mice were challenged with 150 trypomastigotes forms of the Y strain of *T. cruzi*. The parasitemia and survival ratio were monitored daily.

**Figure 5:**
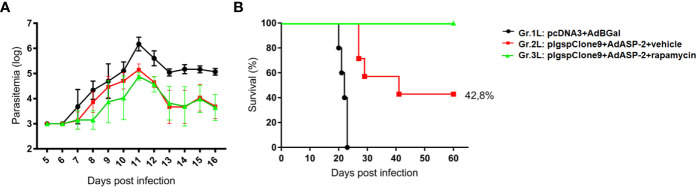
Rapamycin improved the protection of A/Sn mice immunized with reduced doses in experimental challenge with *T. cruzi*. A/Sn mice were immunized *via* i.m. with plasmid (10 µg) and adenovirus (2 x 10^7^ pfu) according to the experimental groups described in the method section. They were also treated daily with rapamycin or vehicle (i.p.) for 34 days. Fifteen days after boosting, mice were infected with 150 blood trypomastigotes of *Y* strain of *T. cruzi*. **(A)** Parasitemia was monitored daily between days 5 and 16 after challenge. The parasitemia values were log transformed and groups 2 and 3 were compared on day 11 (parasitemia peak) by *One-Way* ANOVA and Tukey’s HSD tests (*p<0,0001). **(B)** The survival rate was also followed and analyzed by Log-rank (Mantel-Cox) test (all groups p = 0.0001; groups 2 and 3 p = 0.0218). Results from 7 mice per group.

Parasitemia (**Figure 5A**) was followed from day 5 post infection, and no significant difference were detected between Gr.2L and Gr.3L. Suprisingly, as shown in the survival curve (**Figure 5B**), 42.8% of mice from Gr.2L survived, while the rapamycin-treated mice (Gr.3L) showed 100% of survival rate (Log-rank p = 0.0218). In an independent experiment, rapamycin alone was not able to protect A/Sn mice from the experimental challenge (**Supplementary Figure 6**). These results demonstrate the adjuvant effect of rapamycin when combined with the vaccination, even with 10-fold lower concentrations of plasmid/adenovirus, inducing a protective immune response generated by prime-boost ASP2 immunization.

### Rapamycin Improved the Protective Immune Response Generated by Heterologous Prime-Boost Protocol in A/Sn Mice

To verify whether the CD8^+^ T-cell response in A/Sn mice was also favored by the immunization combined with the rapamycin treatment, we performed the *ex vivo* assays. Splenocytes from A/Sn mice, immunized with 10-fold lower doses and treated with rapamycin or vehicle, were collected on days 35 or 95 after priming to measure the number of specific CD8^+^ cells labeled with the multimer H2K^K^- restricted TEWETGQI peptide. In addition, we performed the intracellular staining to label CD107a, IFNγ and TNF, and ELISpot to measure the number of TEWETGQI-specific IFNγ producing CD8^+^ T-cells.

**Figure 6:**
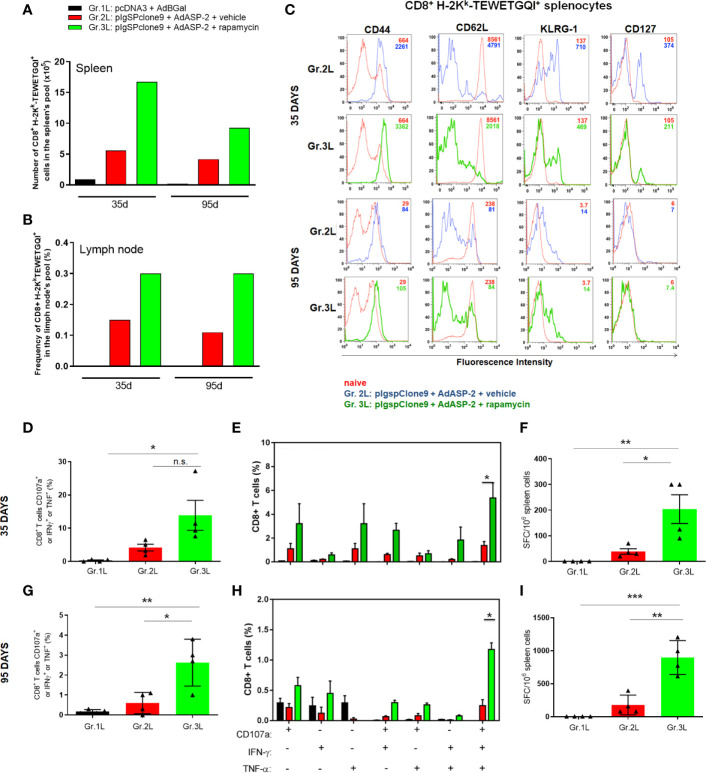
Rapamycin increased the frequency of specific CD8^+^ T cell and immune responses from A/Sn mice, after 35 or 95 days after priming. A/Sn mice were immunized *via* i.m. with plasmid (10 µg) and adenovirus (2 x 10^7^ pfu) according to the experimental groups described in the method section. They were also treated daily with rapamycin or vehicle (i.p.) for 34 days. 35 or 95 days from priming, cells from spleen or lymph nodes were collected for surface staining or *ex vivo* assays. **(A)** Absolut number of specific CD8^+^ T H2K^k^-TEWETGQI^+^ cells from spleen’s pool of each group. **(B)** Frequency of specific CD8^+^ T H2K^k^-TEWETGQI^+^ cells from lymph node’s pool of each group. **(C)** Histograms show the expression of the markers cited above in CD8^+^ H2K^k^-TEWETGQI^+^ cells from Gr.2 (blue lines) and Gr.3 (green lines), or in CD8^+^ H2K^k^-TEWETGQI^-^ from naïve mice (red lines). **(D, G)** CD8^+^ T cell frequencies in percentage expressing CD107a, IFNγ or TNF after stimulation. **(E, H)** Subpopulations of CD8^+^ T cells expressing each individual molecule or combinations (CD107a, IFNγ and/or TNF). **(F, I)** ELISpot assay plots shows mean ± SD of IFNγ producing cells. SFC: Spot-forming cells. Statistical analysis was performed using the *One-Way* ANOVA and Tukey’s HSD tests. Asterisks indicate significant differences among groups, defined as *P < 0.05, **P < 0.01, and ***P < 0.001. Data referring of 2 experiments and 4 mice per group. N.S., Non-significant.

Initially, to quantify TEWETGQI-specific CD8^+^ T-cells, pools of splenocytes and lymph node cells from 4 mice per group were prepared. After labeling the cells with the multimer and anti-CD8, our data show that the frequency and absolute number of TEWETGQI-specific CD8^+^ T-cells increased in the rapamycin-treated mice (Gr.3L) (**Figures 6A, B**). Further, 35 after priming both ASP2-immunized groups (Gr2.L and Gr3L) presented a population of TEWETGQI-specific CD8^+^ T-cells with an activated phenotype, characterized by expression of the cell markers CD44^High^, CD62L^Low^, KLRG1^High^ and CD127^Low^ (**Figure 6C**). At 95 days after priming, the TEWETGQI-specific CD8^+^ T-cells presented a reduced expression of CD127 and KLRG-1 (**Figure 6C**).

At 35 days and 95 days after priming, the frequencies of polyfunctional CD8^+^ T cells (CD107a^+^, IFNγ^+^ and TNF) (**Figures 6D–E, G–H**), and the absolute number of IFNγ^+^ CD8^+^ T cells, as revealed by ELISpot assay (**Figures 6F, I**), were significantly higher in splenocytes obtained from Gr.3L mice, compared to Gr.1L and Gr.2L, after *ex vivo* stimulation with the specific peptide. Altogether, these findings corroborate the improvement of vaccination after rapamycin treatment and explain the protective profile found after the challenge with the virulent *T. cruzi* Y strain, as described above.

### Treatment With Rapamycin Increased *In Vivo* CD8^+^ T-Cell Proliferation

Since the number of ASP2-specific CD8**^+^** T-cells increased in the rapamycin-treated mice (Gr.3 and Gr3L), we hypothesized that CD8**^+^** T-cells might show a differential proliferate response and clonal expansion after activation. Hence, the proliferation of CD8**^+^** T-cells was analyzed *in vivo* using the BrdU incorporation approach. For that, A/Sn mice were immunized and treated with rapamycin or vehicle. After boosting, mice were treated every 48 hours with BrdU (2 mg/dose) until day 35 after priming.

**Figure 7:**
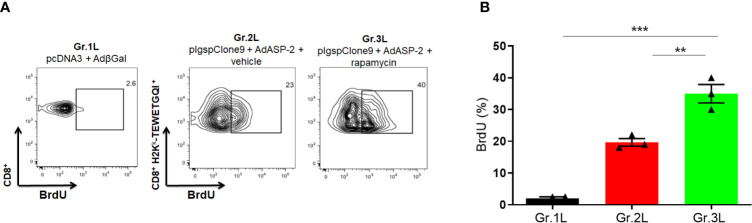
*In vivo* proliferation of specific CD8^+^ T cells was higher in rapamycin-treated group during immunization. A/Sn mice were immunized *via* i.m. with plasmid (10 µg) and adenovirus (2 x 10^7^ pfu) according to the experimental groups described in the method section. They were also treated daily with rapamycin or vehicle (i.p.) for 34 days. Mice were also treated with BrdU after boosting, every 48 hours (2 mg) *via* i.p. **(A)** Fifteen days after boosting, the splenocytes were collected and labeled with anti-CD8, H2K^k^-TEWETGQI^+^ multimer and anti-BrdU to quantify the frequency of incorporating-BrdU cells during immunization. **(B)** Frequencies of BrdU incorporation in CD8^+^ H2K^k^-TEWETGQI^+^ cells with mean ± SD. Statistical analysis was performed using the *One-Way* ANOVA and Tukey’s HSD tests. Asterisks indicate significant differences among groups, defined as **P < 0.01 and ***P < 0.001. Data referring of 3 mice per group.

According to **Figure 7**, ASP2-specific CD8^+^ T-cells from rapamycin-treated mice (Gr.3L) showed a higher incorporation of BrdU compared to Gr.1L and Gr.2L, due to the number of precursors activated by the boost. Therefore, during the expansion phase induced by vaccination, rapamycin treatment potentiated proliferative response of ASP2-specific CD8^+^ T-cells.

### Dendritic Cells Activated in the Presence of Rapamycin Lack Improvement in the Antigenic Presentation Capacity of ASP2-Specific CD8^+^ T-Cells

**Figure 8:**
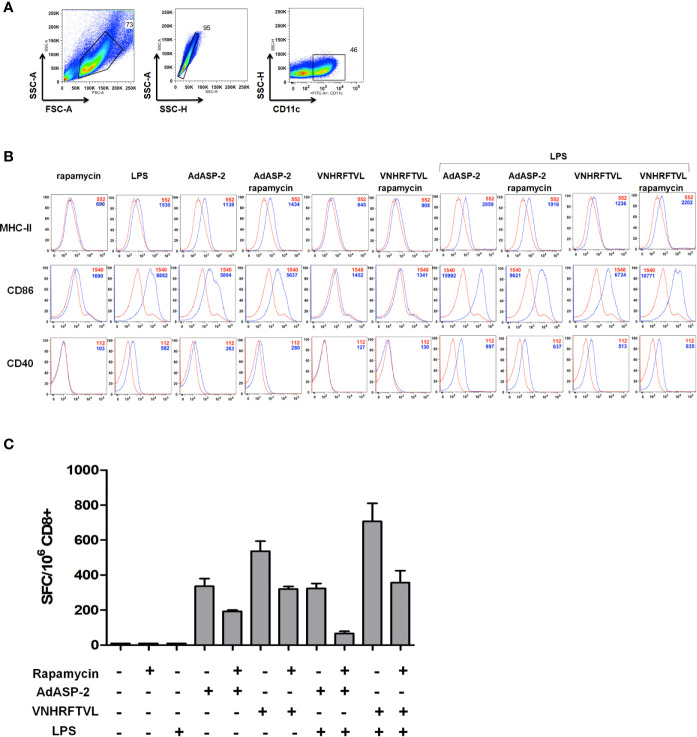
Rapamycin did not affect the MHC-I expression and costimulatory molecules in BMDC, but impaired antigen presentation capacity to CD8^+^ T cells. *In vitro*-generated BMDC from C57BL/6 mice strain were incubated with VNHRFTLV peptide (1 hour), AdASP-2 (24 hours) (blue curves) or left untreated (red curves). Some conditions were treated with rapamycin (1 μM) during stimulation. The same experiment was performed with LPS-primed BMDC (200 ng/mL for 3 hours). The surface expression of CD40, CD86 and MHC class I (H2K^b^) molecules were analyzed by flow cytometry. **(A)** Gate strategy used to select BMDCs CD11c^+^. **(B)** Histograms with the fluorescence intensities (MFI) of the markers in CD11c^+^ cells in unstimulated (red lines) or stimulated (blue lines). **(C)** Purified CD8^+^ T cells were obtained from the spleens of C57BL/6 mice immunized with AdASP-2 (2x10^7^ pfu) 15 days earlier. The purified CD8^+^ T cells were co-cultured with loaded-BMDC and incubated overnight. The frequency of IFNγ-producing cells was detected by ELISpot. The results correspond to the mean ± SD of triplicate values of one experiment. For negative control, CD8^+^ T cells from naive mice were also incubated, but did not produce spots.

Dendritic cells are specialized in antigen processing and presentation, capable of inducing the initial activation of T lymphocytes. The study performed by Amiel et al. (2012) showed that inhibition of mTOR during activation of dendritic cells derived from bone marrow (BMDC) prolonged their useful life and increased the expression of costimulatory molecules, essential for the antigen presentation. In addition, a tuberculosis vaccine (BCG) study that employed the stimulation of dendritic cells in the presence of rapamycin led to an increase in T cell activation (Jagannath et al., 2009). Hence, analyzing the role of dendritic cells antigen presentation for CD8^+^ T-cells during immunization appeared to be important. For that purpose, BMDC of C57BL/6 mice were generated *in vitro* for 7 days, incubated with the ASP2-carrying adenovirus vector (MOI = 50) or the VNHRFTLV peptide (10 μg/ml) in the presence or absence of rapamycin (1 μM). In some conditions, BMDC were primed/matured with LPS (200 ng/mL) for 3 hours. As shown in **Figures 8A, B**, the expression level of costimulatory molecules (CD86 and CD40) and MHC class I (H2K^b^) in CD11c^+^ cells were analyzed. We observed that expression levels of these markers were similar in dendritic cells stimulated in the presence or absence of rapamycin.

Next, we evaluated the antigenic presentation capacity of BMDC stimulated in the presence of rapamycin to activate ASP2-specific CD8^+^ T-cells. Purified CD8^+^ cells from mice previously immunized with AdASP-2 were co-cultured with antigen-loaded BMDC under the same conditions as described above, for 24 hours. The frequency of IFNγ-producing cells was detected by ELISpot. Interestingly, unlike expected, the *in vitro* antigenic presentation by BMDC in the presence of rapamycin decreased CD8^+^ T-cell activation, since the number of IFNγ secreting cells was lower with the VNHRFTLV peptide or AdASP-2 and rapamycin (**Figure 8C**).

## Discussion

The heterologous prime-boost strategy is a well-established protocol capable of generating an effective response by inducing specific CD8^+^ T-cells against *T. cruzi*. The characterization of the phenotype and function of specific CD8^+^ T-lymphocytes show that these cells secrete IFNγ and TNF, express CD107a and are highly cytotoxic *in vivo* (De Alencar et al., 2009). Furthermore, this protocol was able to protect highly susceptible mice in experimental challenges with *T. cruzi* (De Alencar et al., 2009; Dominguez et al., 2011; Rigato et al., 2011). However, we showed here that treatment with rapamycin during immunization was able to potentiate the response of -specific CD8^+^ T-cells.

The number and frequency of immunization-specific CD8^+^ T-cells increased significantly in the rapamycin-treated mice, even after 95 days after priming. It implies that cell contraction was delayed, and rapamycin treatment elicited memory precursor CD8^+^ T-cells rather than short-lived cells. In addition, specific CD8^+^ T-cells generated by immunization and rapamycin treatment showed a TE phenotype after 35 days from priming, and acquire subsequently a TEM profile, which was previously characterized in studies by our group (Rigato et al., 2011). Although rapamycin treatment has been reported to modulate the transition from effector to memory cells, especially to TCM after viral infections and tumors (Araki et al., 2009; Li et al., 2011; Turner et al., 2011; Li et al., 2012; Mattson et al., 2014; Shrestha et al., 2014), this modulation was not detected in our model. However, similar to our results, rapamycin combined with OX-40 stimulation induced a CD8^+^ memory population with a TEM profile following immunization with AdHu5 against LCMV (33), as well as found during the treatment with rapamycin in a carcinoma model (Jung et al., 2018).

The superiority between the TEM and TCM memory profiles is not yet fully established. Although TCM cells have been reported to provide superior long-term protection against systemic infections (Zaph et al., 2004; Klebanoff et al., 2005; Angelosanto and Wherry, 2010), this memory phenotype does not necessarily represent higher quality (Lanzavecchia and Sallusto, 2005). Indeed, CD8^+^ TEM cells generated by heterologous prime-boost immunization protocol confer immunity and protection against *T. cruzi* in acute and chronic infections (De Alencar et al., 2009; Haolla et al., 2009; Rigato et al., 2011; Araújo et al., 2014). TEM CD8^+^ T cells can respond fast during recall and this quality is crucial for protection of individuals in endemic areas.

Additionally, the analysis of the functional response performed by specific CD8^+^ T-cells showed that rapamycin-treated mice had an increased CD8^+^ T response, with greater magnitude and number of polyfunctional cells (IFNγ^+^, TNF^+^, CD107a^+^), especially 95 days after priming. Similar results in CD8^+^ T cell polyfunctionality improvement was found after rapamycin-treatment of rhesus macaques immunized against vaccinia, up to 140 days after immunization, as well as following infection with LCMV (Turner et al., 2011). These results were found here also in mice immunized with 10-fold lower doses of plasmid/adenovirus lasting up to 95 days after priming. Regarding the cytotoxic function, after *in vivo* challenge, CD8^+^ T-cells cytotoxic activity was higher in the rapamycin-treated mice with both peptides concentrations, which enriches the adjuvant potential of rapamycin treatment during the immune challenge of CD8^+^ T-cells, generating functional cells and memory precursors (Araki et al., 2009; Pearce et al., 2009; Mannick et al., 2014; de Souza et al., 2016).

We described for the first time the treatment with rapamycin was able to improve the protection of susceptible A/Sn mice immunized with 10-fold lower doses of plasmid/adenovirus in the experimental challenge with *T. cruzi*. Surprisingly, the rapamycin-treated mice resisted the experimental challenge and showed maximum survival, while only 42.8% of the vehicle-injected mice (Gr.2L) resisted. Immune response assays performed in A/Sn mice also showed that specific CD8^+^ T-cell frequency was higher in the rapamycin-treated group in the spleen and lymph node, as well as the production of IFNγ and the number of polyfunctional cells. Taken together, these results strongly confirm the positive effect of rapamycin during immunization, as differences were found in distinct mouse strains up to 95 days after the priming.

Related to the increase in specific CD8^+^ T population, the results obtained here suggest that rapamycin induced a higher proliferation rate of specific CD8^+^ T-cells during differentiation and expansion phase, which occurs concurrently with *in vivo* rapamycin treatment. Although it was reported by Araki (Araki et al., 2009) that after 30 days of viral challenge and rapamycin treatment there was minimal incorporation of BrdU into DNA of specific CD8^+^ T-cells in all groups, showing that the decrease in T-cell contraction was not due to increase of cell proliferation but probably by survival, our data indicate that modulation exerted by rapamycin may inhibit cell contraction by different mechanisms, depending on the infection model and vaccine protocol used.

By investigating the effect of rapamycin on dendritic cells and how it would influence cellular response, the treatment suppressed CD8^+^ T-cell presentation and activation ability. This result corroborates previous studies showing that inhibition of mTOR appears to suppress DCs differentiation and maturation, so the cells exposed to rapamycin have an impaired ability to stimulate T-cells and cytokine production (Hackstein et al., 2003; Araki et al., 2010). Another *in vitro* study showed a suppressive effect on some aspects of DCs function at high doses of rapamycin, while at decreasing doses this effect was reversed, promoting the inflammatory function of cytokines (Gammon et al., 2017). Taken together, these data suggest that mTOR signaling has several effects on both inhibitory and stimulatory dendritic cells.

It is important to mention that our results presented some limitations, including the small sample size used in both *in vivo* and *in vitro* experiments as well as the use of splenocyte’s pools instead of individual splenocytes in the multimer experiments with A/Sn mice. Despite that, our findings are exciting and open new approaches to understand and validate the modulation made by rapamycin in vaccine context.

By knowing this, it is not clear how the blockage by rapamycin and mTOR pathways may interact synergistically to improve CD8^+^ T-cell memory. Several published studies have demonstrated that autophagy and metabolic switches are important for memory development (Araki et al., 2009; Pearce et al., 2009; Jagannath and Bakhru, 2012; Puleston et al., 2014; Xu et al., 2014; Chang and Pearce, 2016). Following activation, CD8^+^ T-cells increase their glucose uptake and produce ATP by glycolysis through mTORC1 signaling. After the contraction phase, the memory population acquires a catabolic metabolism based on oxidative phosphorylation by the oxidation of fatty acids (FAO) and amino acids (Jones and Thompson, 2007). Pearce (Pearce et al., 2009) demonstrated that fat acid oxidation regulates memory development of CD8^+^ T-cells. Additionally, autophagy has been also described as important to memory formation of CD8^+^ T-cells due to the molecules recycling, damage repairing and product substrates for oxidative phosphorylation and FAO (Puleston et al., 2014; Xu et al., 2014). Since activation and other cellular processes lead to activation of mTOR, which induces glycolysis to support cell growth, proliferation and cell function (Chang and Pearce, 2016), it will be necessary to examine whether mTOR inhibition during immunization could interfere with T cell metabolism and bioenergetic capacity, polarizing the response to a metabolic profile similar to long-term memory cells.

The induction of memory cells is important due to long-lasting persistence, but also due to the ability to respond in the antigen recall. A catabolic capacity and greater mitochondrial mass confer the ability to respond fast and powerfully against the antigen, providing long-lasting protection (van der Windt et al., 2013). Therefore, the interest in generating a functional memory population through modulating their metabolic profile and energy capacity, will have more concern in the vaccine development field against chronic diseases and tumors.

Although rapamycin inhibition of mTOR has been reported to impair the differentiation of effector CD8^+^ T-cells, lead to loss of function, failure to control viral infections and cellular anergy (Araki et al., 2010; Yao et al., 2013; Goldberg et al., 2014), this was not observed in this study. Indeed, it was evident that treatment with rapamycin in our vaccination model does not impair the differentiation of the specific CD8^+^ T population, cytokine production and cytotoxic activity, both in the effector and memory phases, which culminates in increased protection after challenge with *T. cruzi*.

Importantly, the dose of rapamycin used in our study and others cited was suboptimal, since low doses were used compared to treatments aimed an immunosuppression effect (Araki et al., 2009; Araki et al., 2010; Gammon et al., 2017). *In vivo* drug’s administration was unable to completely block mTOR signaling and this inhibition occurs in a dose-dependent manner (Araki et al., 2009; Gammon et al., 2017), as observed in CD8^+^ T-cells when mTOR expression was decreased by RNAi, suggesting that rapamycin stimulates the formation of memory CD8^+^ T-cells by incompletely inhibition of mTOR signaling. Higher doses of rapamycin may result in suppression of CD8^+^ T cell expansion (Araki et al., 2009).

Given that mTOR signaling plays a central role in regulating cellular responses, this appears to be a potential pathway to be explored for modulating immune responses induced by vaccines (Sallusto et al., 2010). Here, we have shown for the first time the use of rapamycin improves functional qualities as well as the frequency of specific CD8^+^ T-cells generated by heterologous prime-boost immunization against *T. cruzi*. This adjuvant effect was seen during the effector and memory phase, even with low immunogenic doses induced by the vaccination, besides promoting protection after experimental challenge. We speculate that mTOR inhibition by rapamycin is acting synergistically on CD8^+^ T-cells, modulating their activation, proliferation, differentiation and possibly their metabolism. Although some mechanisms need to be further elucidated, these findings suggest that strategic rapamycin treatment may improve effector and memory cell development in response to vaccine protocols, offering a new method for adjusting the desired immune response as well as plasticity, phenotype and cellular function.

In order to induce a population with a phenotype that provides optimal protection and functional qualities of memory CD8^+^ T-cells against the target pathogen, we provided herein a novel approach to upgrade the efficacy of genetic vaccines against intracellular infections, such as Chagas disease.

## Data Availability Statement

The raw data supporting the conclusions of this article will be made available by the authors, without undue reservation.

## Ethics Statement

The animal study was reviewed and approved by Ethical Committee for Animal Experimentation at the Federal University of Sao Paulo, registered under number 9959021014.

## Author Contributions

BM, MR and JV conceived and designed the experiments. BM, IN, CF, LC, CM, PD, and TV performed the experiments. BM, IN, CF and JV analyzed the experiment data. RG, JL-V, KB, GP and JV contributed with reagents and materials, and additional experiments design. BM wrote the manuscript. BM, JL-V, KB and JV reviewed the manuscript. All authors contributed to the article and approved the submitted version.

## Funding

This work was supported by grants from Fundação de Amparo à Pesquisa do Estado de São Paulo (http://www.fapesp.br/) (BM: 2014/19422-5, 2016/02840-4; CF: 2015/08814-2; JV: 2012/22514-3, 2018/15607-1), Instituto Nacional de Ciência e Tecnologia em Vacinas (http://inct.cnpq.br/), Coordenação de Aperfeiçoamento de Pessoal de Nível Superior (http://www.capes.gov.br/) and Conselho Nacional de Desenvolvimento Científico e Tecnológico (http://cnpq.br/).

## Conflict of Interest

The authors declare that the research was conducted in the absence of any commercial or financial relationships that could be construed as a potential conflict of interest.

## Supplementary Material

Supplementary Figure 1 Expression of Ribosomal Phospho-S6 (PS6) protein in CD8^+^ T cells after treatment with rapamycin. Splenocytes from naïve mice were incubated with Concavalin A (2 mg/mL) or rapamycin (100 nM) for one hour. Then, cells were stained with anti-CD8, fixed and permeabilizated to anti-pS6K staining. **(A)** Dot-plots show the frequency of pS6K in singlets in each condition. **(B)** Histograms indicate the mean of fluorescence intensity of cells in medium (red line), treated with rapamycin (green line) or stimulated with ConA (blue line).

Supplementary Figure 2 Strategy used for analysis of intracellular staining of cytokines in CD8^+^ T cells. Splenocytes were treated conform described in the methods section. **(A)** Gate strategy used for positive selection of CD8^+^ cells. **(B)** Dot-plots represent the frequencies of CD107a, IFNg and TNF in CD8^+^ T cells from immunized mice after stimulation with the specific peptide in vitro. Data correspondent to a representative mouse.

Supplementary Figure 3 Immunophenotyping of specific CD8^+^ T cells from C57BL/6 mice immunized and treated with rapamycin or diluent after 35 days from priming. C57BL/6 mice were immunized via i.m. with plasmid (100 µg) and adenovirus (2 x 10^8^ pfu) according to the experimental groups described in the method section. They were also treated daily with rapamycin or vehicle (i.p.) for 34 days. After 35 days from priming, splenocytes were labeled with anti-CD8, H2K^b^-VNHRFTLV multimer and with the specific markers indicated above for flow cytometric analysis. Histograms show the expression of the markers in CD8^+^ H2K^b^-VNHRFTLV^+^ cells (blue and green lines) or CD8^+^ cells of naive as control (red lines). Analyses were performed using cells pools of 4 mice and they are representative of two independent experiments. The numbers indicate the mean fluorescence intensity (MFI). The individual analysis of mice from each group presented similar results.

Supplementary Figure 4 Immunophenotyping of specific CD8^+^ T cells of C57BL/6 mice immunized and treated with rapamycin or diluent after 95 from priming. C57BL/6 mice were immunized *via* i.m. with plasmid (100 µg) and adenovirus (2 x 10^8^ pfu) according to the experimental groups described in the method section. They were also treated daily with rapamycin or vehicle (i.p.) for 34 days. After 35 days from priming, splenocytes were labeled with anti-CD8, H2K^b^-VNHRFTLV multimer and with the specific markers indicated above for flow cytometric analysis. Histograms show the expression of the markers in CD8^+^ H2K^b^-VNHRFTLV^+^ cells (blue and green lines) or CD8^+^ cells of naive as control (red lines). Analyses were performed using cells pools of 4 mice and they are representative of two independent experiments. The numbers indicate the mean fluorescence intensity (MFI). The individual analysis of mice from each group presented similar results.

Supplementary Figure 5 In vivo cytotoxicity of specific CD8^+^ T cells of C57BL/6 mice immunized and treated with rapamycin or diluent. C57BL/6 mice were immunized *via* i.m. with plasmid (100 µg) and adenovirus (2 x 10^8^ pfu) according to the experimental groups described in the method section. They were also treated daily with rapamycin or vehicle (i.p.) for 34 days. Splenocytes from naive mice were stained with CFSE in 2 different concentrations. CFSE^High^ population was pulsed with peptide VNHRFTLV at a final concentration of 2 mM. CFSE^Low^ was the negative control. Stained cells were transferred to the experimental groups and, after 14 hours, spleens were harvested to quantify the frequency of stained cells. **(A)** Histograms represent the frequencies of CFSE^High^ and CFSE^Low^ in each group. **(B)** Percentage of CD8^+^ T mediated cytotoxicity, with mean ± SD. Results from one experiment and 4 mice per group. Statistical analysis was performed using the One-Way ANOVA and Tukey’s HSD tests. Asterisks indicate significant differences among groups, defined as *P < 0.05, **P < 0.01, and ***P < 0.001. N.S., Non-significant.

Supplementary Figure 6 Experimental challenge of A/sn mice after treatment with rapamycin or vehicle. A/Sn mice were treated daily with rapamycin or vehicle (PBS) for 34 days. On the last day, mice were infected with 150 blood trypomastigotes of Y strain of T. cruzi. **(A)** Parasitemia was monitored daily between days 6 and 15 after challenge. The parasitemia values were log transformed. **(B)** The survival rate was also followed and analyzed by Log-rank (Mantel-Cox) test (all groups p = 0,0285). Results from 4 mice per group.

## References

[B1] Ahmed R. Akondy R. S. (2011). Insights Into Human CD8+ T-Cell Memory Using the Yellow Fever and Smallpox Vaccines. Immunol. Cell Biol. 89, 340–345. 10.1038/icb.2010.155 21301482

[B2] Amiel E. Everts B. Freitas T. C. King I. L. Curtis J. D. Pearce E. L.. (2012). Inhibition of Mechanistic Target of Rapamycin Promotes Dendritic Cell Activation and Enhances Therapeutic Autologous Vaccination in Mice. J. Immunol 189 (5), 2151–2158. 10.4049/jimmunol.1103741 22826320 PMC3424310

[B3] Angelosanto J. M. Wherry E. J. (2010). Transcription Factor Regulation of CD8+ T-Cell Memory and Exhaustion. Immunol. Rev. 236, 167–175. 10.1111/j.1600-065X.2010.00927.x 20636816

[B4] Araki K. Turner A. P. Shaffer V. O. Gangappa S. Keller S. A. Bachmann M. F.. (2009). mTOR Regulates Memory CD8 T-Cell Differentiation. Nature 460, 108–112. 10.1038/nature08155 19543266 PMC2710807

[B5] Araki K. Youngblood B. Ahmed R. (2010). The Role of mTOR in Memory CD8+ T-Cell Differentiation. Immunol. Rev. 235 (1), 234–243. 10.1111/j.0105-2896.2010.00898.x 20536567 PMC3760155

[B6] Araújo A. F. de Oliveira G. Vasconcelos J. F. Ersching J. Dominguez M. R. Vasconcelos J. R.. (2014). Genetic Vaccination Against Experimental Infection With Myotropic Parasite Strains of Trypanosoma Cruzi. Mediators Inflamm. 2014, 1–13. 10.1155/2014/605023 PMC4098640 25061263

[B7] Augustine J. J. Bodziak K. A. Hricik D. E. (2007). Use of Sirolimus in Solid Organ Transplantation. Drugs 67 (3), 369–391. 10.2165/00003495-200767030-00004 17335296

[B8] Bassett J. D. Swift S. L. Vanseggelen H. Hammill J. A. McGray A. R. Evelegh C.. (2012). Combined mTOR Inhibition and OX40 Agonism Enhances CD8 T Cell Memory and Protective Immunity Produced by Recombinant Adenovirus Vaccines. Mol. Ther. 20, 860–869. 10.1038/mt.2011.281 22186790 PMC3321597

[B9] Borsa M. Barnstorf I. Baumann N. S. Pallmer K. Yermanos A. Gräbnitz F.. (2019). Modulation of Asymmetric Cell Division as a Mechanism to Boost CD8+ T Cell Memory. Sci. Immunol. 4 (34), eaav1730. 10.1126/sciimmunol.aav1730 30979796

[B10] Buck M. D. O’Sullivan D. Pearce E. L. (2015). T Cell Metabolism Drives Immunity. J. Exp. Med. 212 (9), 1345–1360. 10.1084/jem.20151159 26261266 PMC4548052

[B11] Chang C. H. Pearce E. L. (2016). Emerging Concepts of T Cell Metabolism as a Target of Immunotherapy. Nat. Immunol. 17 (4), 364–368. 10.1038/ni.3415 27002844 PMC4990080

[B12] Chuang I. Sedegah M. Cicatelli S. Spring M. Polhemus M. Tamminga C.. (2013). Dna Prime/Adenovirus Boost Malaria Vaccine Encoding P. Falciparum CSP and AMA1 Induces Sterile Protection Associated With Cell-Mediated Immunity. PloS One 8 (2), e55571. 10.1371/journal.pone.0055571 23457473 PMC3573028

[B13] Cui W. Kaech S. M. (2010). Generation of Effector CD8+ T Cells and Their Conversion to Memory T Cells. Immunol. Rev. 236, 151–166. 10.1111/j.1600-065X.2010.00926.x 20636815 PMC4380273

[B14] De Alencar B. C. G. Persechini P. M. Haolla F. A. De Oliveira G. Silverio J. C. Lannes-Vieira J.. (2009). Perforin and Gamma Interferon Expression are Required for CD4+ and CD8+ T-Cell-Dependent Protective Immunity Against a Human Parasite, Trypanosoma Cruzi, Elicited by Heterologous Plasmid DNA Prime-Recombinant Adenovirus 5 Boost Vaccination. Infect. Immun. 77, 4383–4395. 10.1128/IAI.01459-08 19651871 PMC2747960

[B15] Dennis P. B. Jaeschke A. Saitoh M. Fowler B. Kozma S. C. Thomas G. (2001). Mammalian TOR: A Homeostatic ATP Sensor. Sci. (80- ) 294 (5544), 1102–1105. 10.1126/science.1063518 11691993

[B16] de Souza A. P. D. de Freitas D. N. Antuntes Fernandes K. E. D’Avila da Cunha M. Antunes Fernandes J. L. Benetti Gassen R.. (2016). Respiratory Syncytial Virus Induces Phosphorylation of mTOR At ser2448 in CD8 T Cells From Nasal Washes of Infected Infants. Clin. Exp. Immunol. 183, 248–257. 10.1111/cei.12720 26437614 PMC4711155

[B17] Dominguez M. R. Silveira E. L. V. de Vasconcelos J. R. C. de Alencar B. C. G. Machado A. V. Bruna-Romero O.. (2011). Subdominant/Cryptic CD8 T Cell Epitopes Contribute to Resistance Against Experimental Infection With a Human Protozoan Parasite. PloS One 6, e22011. 10.1371/journal.pone.0022011 21779365 PMC3136500

[B18] Elvang T. Christensen J. P. Billeskov R. Thi Kim Thanh Hoang T. Holst P. Thomsen A. R.. (2009). CD4 and CD8 T Cell Responses to the M. Tuberculosis Ag85B-TB10.4 Promoted by Adjuvanted Subunit, Adenovector or Heterologous Prime Boost Vaccination. PloS One 4, e5139. 10.1371/journal.pone.0005139 19357780 PMC2663846

[B19] Ersching J. Basso A. S. Kalich V. L. G. Bortoluci K. R. Rodrigues M. M. (2016). A Human Trypanosome Suppresses CD8+ T Cell Priming by Dendritic Cells Through the Induction of Immune Regulatory CD4+ Foxp3+ T Cells. PloS Pathog. 12, 1–23. 10.1371/journal.ppat.1005698 PMC4917094 27332899

[B20] Ersching J. Efeyan A. Mesin L. Jacobsen J. T. Pasqual G. Grabiner B. C.. (2017). Germinal Center Selection and Affinity Maturation Require Dynamic Regulation of mTORC1 Kinase. Immunity 46, 1045–1058.e6. 10.1016/j.immuni.2017.06.005 28636954 PMC5526448

[B21] Ferreira C. P. Cariste L. M. Virgílio F. D. S. Moraschi B. F. Monteiro C. B. Machado A. M. V.. (2017). LFA-1 Mediates Cytotoxicity and Tissue Migration of Specific CD8+ T Cells After Heterologous Prime-Boost Vaccination Against Trypanosoma Cruzi Infection. Front. Immunol. 8, 1291. 10.3389/fimmu.2017.01291 29081775 PMC5645645

[B22] Gammon J. M. Gosselin E. A. Tostanoski L. H. Chiu Y. C. Zeng X. Zeng Q.. (2017). Low-Dose Controlled Release of mTOR Inhibitors Maintains T Cell Plasticity and Promotes Central Memory T Cells. J. Control Release 263, 151–161. 10.1016/j.jconrel.2017.02.034 28257991 PMC5573661

[B23] Gilbert S. C. Schneider J. Hannan C. M. Hu J. T. Plebanski M. Sinden R.. (2002). Enhanced CD8 T Cell Immunogenicity and Protective Efficacy in a Mouse Malaria Model Using a Recombinant Adenoviral Vaccine in Heterologous Prime-Boost Immunisation Regimes. Vaccine 20, 1039–1045. 10.1016/S0264-410X(01)00450-9 11803063

[B24] Goldberg E. L. Smithey M. J. Lutes L. K. Uhrlaub J. L. Nikolich-Žugich J. (2014). Immune Memory–Boosting Dose of Rapamycin Impairs Macrophage Vesicle Acidification and Curtails Glycolysis in Effector Cd8 Cells, Impairing Defense Against Acute Infections. J. Immunol. 193 (2), 757–763. 10.4049/jimmunol.1400188 24913978 PMC4119820

[B25] Graham S. P. McLean R. K. Spencer A. J. Belij-Rammerstorfer S. Wright D. Ulaszewska M.. (2020). Evaluation of the Immunogenicity of Prime-Boost Vaccination With the Replication-Deficient Viral Vectored COVID-19 Vaccine Candidate ChAdOx1 Ncov-19. NPJ Vaccines 5 (1), 69. 10.1038/s41541-020-00221-3 PMC7385486 32793398

[B26] Hackstein H. Taner T. Zahorchak A. F. Morelli A. E. Logar A. J. Gessner A.. (2003). Rapamycin Inhibits IL-4-Induced Dendritic Cell Maturation In Vitro and Dendritic Cell Mobilization and Function In Vivo. Blood 101 (11), 4457–4463. 10.1182/blood-2002-11-3370 12531798

[B27] Haolla F. A. Claser C. de Alencar B. C. G. Tzelepis F. de Vasconcelos J. R. de Oliveira G.. (2009). Strain-Specific Protective Immunity Following Vaccination Against Experimental Trypanosoma Cruzi Infection. Vaccine 27, 5644–5653. 10.1016/j.vaccine.2009.07.013 19635607

[B28] Hensley L. E. Mulangu S. Asiedu C. Johnson J. Honko A. N. Stanley D.. (2010). Demonstration of Cross-Protective Vaccine Immunity Against an Emerging Pathogenic Ebolavirus Species. PloS Pathog. 6 (5), e1000904. 10.1371/journal.ppat.1000904 20502688 PMC2873919

[B29] Hill A. V. S. Reyes-Sandoval A. O’Hara G. Ewer K. Lawrie A. Goodman A.. (2010). Prime-Boost Vectored Malaria Vaccines: Progress and Prospects. Hum. Vaccin 6, 78–83. 10.4161/hv.6.1.10116 20061802

[B30] Jacobs S. R. Herman C. E. MacIver N. J. Wofford J. A. Wieman H. L. Hammen J. J.. (2008). Glucose Uptake is Limiting in T Cell Activation and Requires Cd28-Mediated Akt-Dependent and Independent Pathways. J. Immunol. 180 (7), 4476–4486. 10.4049/jimmunol.180.7.4476 18354169 PMC2593791

[B31] Jagannath C. Bakhru P. (2012). Rapamycin-Induced Enhancement of Vaccine Efficacy in Mice. Methods Mol. Biol. 821, 295–303. 10.1007/978-1-61779-430-8_18 22125073 PMC3387998

[B32] Jagannath C. Lindsey D. R. Dhandayuthapani S. Xu Y. Hunter R. L. Eissa N. T. (2009). Autophagy Enhances the Efficacy of BCG Vaccine by Increasing Peptide Presentation in Mouse Dendritic Cells. Nat. Med. 15 (3), 267–276. 10.1038/nm.1928 19252503

[B33] Jones R. G. Thompson C. B. (2007). Revving the Engine: Signal Transduction Fuels T Cell Activation. Immunity 27 (2), 173–178. 10.1016/j.immuni.2007.07.008 17723208

[B34] Jung J. W. Veitch M. Bridge J. A. Overgaard N. H. Cruz J. L. Linedale R.. (2018). Clinically-Relevant Rapamycin Treatment Regimens Enhance CD8+ Effector Memory T Cell Function in The Skin and Allow Their Infiltration Into Cutaneous Squamous Cell Carcinoma. Oncoimmunology 7 (9), e1479627. 10.1080/2162402X.2018.1479627 30228949 PMC6140608

[B35] Klebanoff C. A. Waldmann T. A. Palmer D. C. Torabi-Parizi P. Restifo N. P. Cardones A. R.. (2005). Central Memory Self/Tumor-Reactive CD8+ T Cells Confer Superior Antitumor Immunity Compared With Effector Memory T Cells. Proc. Natl. Acad. Sci. 102, 9571–9576. 10.1073/pnas.0503726102 15980149 PMC1172264

[B36] Lanzavecchia A. Sallusto F. (2005). Understanding the Generation and Function of Memory T Cell Subsets. Curr. Opin. Immunol. 17, 326–332. 10.1016/j.coi.2005.04.010 15886125

[B37] Li X. Garcia K. Sun Z. Xiao Z. (2011). Temporal Regulation of Rapamycin on Memory CTL Programming by IL-12. PloS One 6 (9), e25177. 10.1371/journal.pone.0025177 21966447 PMC3179471

[B38] Li Q. Rao R. Vazzana J. Goedegebuure P. Odunsi K. Gillanders W.. (2012). Regulating mTOR to Tune Vaccination Induced CD8+ T Cell Responses for Tumor Immunity. J. Immunol. 188, 3080–3087. 10.4049/jimmunol.1103365.Regulating 22379028 PMC3311730

[B39] Li S. Rodrigues M. Rodriguez D. Rodriguez J. R. Esteban M. Palese P.. (1993). Priming With Recombinant Influenza Virus Followed by Administration of Recombinant Vaccinia Virus Induces CD8+ T-Cell-Mediated Protective Immunity Against Malaria. Proc. Natl. Acad. Sci. 90 (11), 5214–5218. 10.1073/pnas.90.11.5214 7685119 PMC46686

[B40] Mannick J. B. Del Giudice G. Lattanzi M. Valiante N. M. Praestgaard J. Huang B.. (2014). mTOR Inhibition Improves Immune Function in the Elderly. Sci. Transl. Med. 6 (268), 268ra179. 10.1126/scitranslmed.3009892 25540326

[B41] Mannick J. B. Morris M. Hockey H. U. Roma G. Beibel M. Kulmatycki K.. (2018). TORC1 Inhibition Enhances Immune Function and Reduces Infections in the Elderly. Sci. Transl. Med. 10 (449), eaaq1564. 10.1126/scitranslmed.aaq1564 29997249

[B42] Martins M. A. Wilson N. A. Reed J. S. Ahn C. D. Klimentidis Y. C. Allison D. B.. (2010). T-Cell Correlates of Vaccine Efficacy After a Heterologous Simian Immunodeficiency Virus Challenge. J. Virol 84 (9), 4352–4365. 10.1128/jvi.02365-09 20164222 PMC2863752

[B43] Mattson E. Xu L. Li L. Liu G. E. Xiao Z. (2014). Transcriptome Profiling of CTLs Regulated by Rapamycin Using RNA-Seq. Immunogenetics 66, 625–633. 10.1007/s00251-014-0790-5 25113844 PMC4198470

[B44] McConkey S. J. Reece W. H. H. Moorthy V. S. Webster D. Dunachie S. Butcher G.. (2003). Enhanced T-cell Immunogenicity of Plasmid DNA Vaccines Boosted by Recombinant Modified Vaccinia Virus Ankara in Humans. Nat. Med. 9, 729–735. 10.1038/nm881 12766765

[B45] Nam J. H. (2009). Rapamycin: Could it Enhance Vaccine Efficacy? Expert Rev. Vaccines 8, 1535–1539. 10.1586/erv.09.115 19863245

[B46] Pearce E. L. Walsh M. C. Cejas P. J. Harms G. M. Shen H. Wang L. S.. (2009). Enhancing CD8 T-Cell Memory by Modulating Fatty Acid Metabolism. Nature 460 (7251), 103–107. 10.1038/nature08097 19494812 PMC2803086

[B47] Pérez-Molina J. A. Molina I. (2017). Chagas Disease. Lancet 6736, 1–13. 10.1016/S0140-6736(17)31612-4 28673423

[B48] Powell J. D. Pollizzi K. N. Heikamp E. B. Horton M. R. (2012). Regulation of Immune Responses by Mtor. Annu. Rev. Immunol. 30, 39–68. 10.1146/annurev-immunol-020711-075024 22136167 PMC3616892

[B49] Puleston D. J. Zhang H. Powell T. J. Lipina E. Sims S. Panse I.. (2014). Autophagy is a Critical Regulator of Memory CD8(+) T Cell Formation. Elife 3, e03706. 10.7554/eLife.03706 PMC4225493 25385531

[B50] Ranasinghe C. Ramshaw I. A. (2009). Genetic Heterologous Prime-Boost Vaccination Strategies for Improved Systemic and Mucosal Immunity. Expert Rev. Vaccines 8, 1171–1181. 10.1586/erv.09.86 19722891

[B51] Rao R. R. Li Q. Odunsi K. Shrikant P. A. (2010). The Mtor Kinase Determines Effector Versus Memory Cd8 + T Cell Fate by Regulating the Expression of Transcription Factors T-bet and Eomesodermin. Immunity 32 (1), 67–78. 10.1016/j.immuni.2009.10.010 20060330 PMC5836496

[B52] Rigato P. O. de Alencar B. C. de Vasconcelos J. R. C. Dominguez M. R. Araújo A. F. Machado A. V.. (2011). Heterologous Plasmid Dna Prime-Recombinant Human Adenovirus 5 Boost Vaccination Generates a Stable Pool of Protective Long-Lived CD8+ T Effector Memory Cells Specific for a Human Parasite, Trypanosoma Cruzi. Infect. Immun. 79, 2120–2130. 10.1128/iai.01190-10 21357719 PMC3088135

[B53] Sallusto F. Lanzavecchia A. Araki K. Ahmed R. (2010). From Vaccines to Memory and Back. Immunity 33 (4), 451–463. 10.1016/j.immuni.2010.10.008 21029957 PMC3760154

[B54] Sheridan B. S. Lefrançois L. (2011). Regional and Mucosal Memory T Cells. Nat. Immunol. 12, 485–491. 10.1038/ni.2029 21739671 PMC3224372

[B55] Shrestha S. Yang K. Wei J. Karmaus P. W. F. Neale G. Chi H. (2014). Tsc1 Promotes the Differentiation of Memory CD8+ T Cells Via Orchestrating the Transcriptional and Metabolic Programs. Proc. Natl. Acad. Sci. U S A 111, 14858–14863. 10.1073/pnas.1404264111 25271321 PMC4205612

[B56] Thomson A. W. Turnquist H. R. Raimondi G. (2009). Immunoregulatory Functions of mTOR Inhibition. Nat. Rev. Immunol 9 (5), 324–337. 10.1038/nri2546 19390566 PMC2847476

[B57] Turner A. P. Shaffer V. O. Araki K. Martens C. Turner P. L. Gangappa S.. (2011). Sirolimus Enhances the Magnitude and Quality of Viral-Specific CD8 + T-cell Responses to Vaccinia Virus Vaccination in Rhesus Macaques. Am. J. Transplant 11, 613–618. 10.1111/j.1600-6143.2010.03407.x 21342450 PMC3076606

[B58] van der Windt G. J. W. O’Sullivan D. Everts B. Huang S. C.-C. Buck M. D. Curtis J. D.. (2013). CD8 Memory T Cells Have a Bioenergetic Advantage That Underlies Their Rapid Recall Ability. Proc. Natl. Acad. Sci. 110, 14336–14341. 10.1073/pnas.1221740110 23940348 PMC3761631

[B59] Vasconcelos J. R. Dominguez M. R. Araújo A. F. Ersching J. Tararam C. A. Bruna-Romero O.. (2012). Relevance of Long-Lived CD8+T Effector Memory Cells for Protective Immunity Elicited by Heterologous Prime-Boost Vaccination. Front. Immunol. 3, 1–11. 10.3389/fimmu.2012.00358 23264773 PMC3525016

[B60] Wherry E. J. Teichgräber V. Becker T. C. Masopust D. Kaech S. M. Antia R.. (2003). Lineage Relationship and Protective Immunity of Memory CD8T Cell Subsets. Nat. Immunol. 4, 225–234. 10.1038/ni889 12563257

[B61] Wilson N. A. Reed J. Napoe G. S. Piaskowski S. Szymanski A. Furlott J.. (2006). Vaccine-Induced Cellular Immune Responses Reduce Plasma Viral Concentrations After Repeated Low-Dose Challenge With Pathogenic Simian Immunodeficiency Virus Sivmac239. J. Virol 80 (12), 5875–5885. 10.1128/jvi.00171-06 16731926 PMC1472612

[B62] Wirth T. C. Badovinac V. P. Zhao L. Dailey M. O. Harty J. T. (2009). Differentiation of Central Memory CD8 T Cells is Independent of CD62L-Mediated Trafficking to Lymph Nodes. J. Immunol. 182, 6195–6206. 10.4049/jimmunol.0803315 19414773

[B63] Wirth T. C. Xue H.-H. Rai D. Sabel J. T. Bair T. Harty J. T.. (2010). Repetitive Antigen Stimulation Induces Stepwise Transcriptome Diversification But Preserves a Core Signature of Memory CD8(+) T Cell Differentiation. Immunity 33, 128–140. 10.1016/j.immuni.2010.06.014 20619696 PMC2912220

[B64] World Health Organization. (2016). Chagas Disease (American trypanosomiasis). World Health Organization. Available from: http://www.who.int/chagas/epidemiology/en/. PMC2305660 10063697

[B65] Wullschleger S. Loewith R. Hall M. N. (2006). TOR Signaling in Growth and Metabolism. Cell 124 (3), 471–484. 10.1016/j.cell.2006.01.016 16469695

[B66] Xu X. Araki K. Li S. Han J. H. Ye L. Tan W. G.. (2014). Autophagy is Essential for Effector CD8 + T Cell Survival and Memory Formation. Nat. Immunol 15 (12), 1152–1161. 10.1038/ni.3025 25362489 PMC4232981

[B67] Yao S. Buzo B. F. Pham D. Jiang L. Taparowsky E. J. Kaplan M. H.. (2013). Interferon Regulatory Factor 4 Sustains CD8+ T Cell Expansion and Effector Differentiation. Immunity. 10.1016/j.immuni.2013.10.007 PMC3855863 24211184

[B68] Zaph C. Uzonna J. Beverley S. M. Scott P. (2004). Central Memory T Cells Mediate Long-Term Immunity to Leishmania Major in the Absence of Persistent Parasites. Nat. Med. 10, 1104–1110. 10.1038/nm1108 15448686

[B69] Zavala F. Rodrigues M. Rodriguez D. Rodriguez J. R. Nussenzweig R. S. Esteban M. (2001). A Striking Property of Recombinant Poxviruses: Efficient Inducers of In Vivo Expansion of Primed CD8+ T Cells. Virology 280 (2), 155–159. 10.1006/viro.2000.0792 11162829

[B70] Zhang G. Huong V. T. T. Battur B. Zhou J. Zhang H. Liao M.. (2007). A Heterologous Prime-Boost Vaccination Regime Using DNA and a Vaccinia Virus, Both Expressing GRA4, Induced Protective Immunity Against Toxoplasma Gondii Infection in Mice. Parasitology 134, 1339. 10.1017/S0031182007002892 17506929

