# Supplementary figures and images for: Rapamycin Improves the Response of Effector and Memory CD8+ T Cells Induced by Immunization With ASP2 of Trypanosoma cruzi
Source: Front Cell Infect Microbiol. 2021 May 25;11:676183. doi: 10.3389/fcimb.2021.676183 (PMC8191465; doi:10.3389/fcimb.2021.676183)

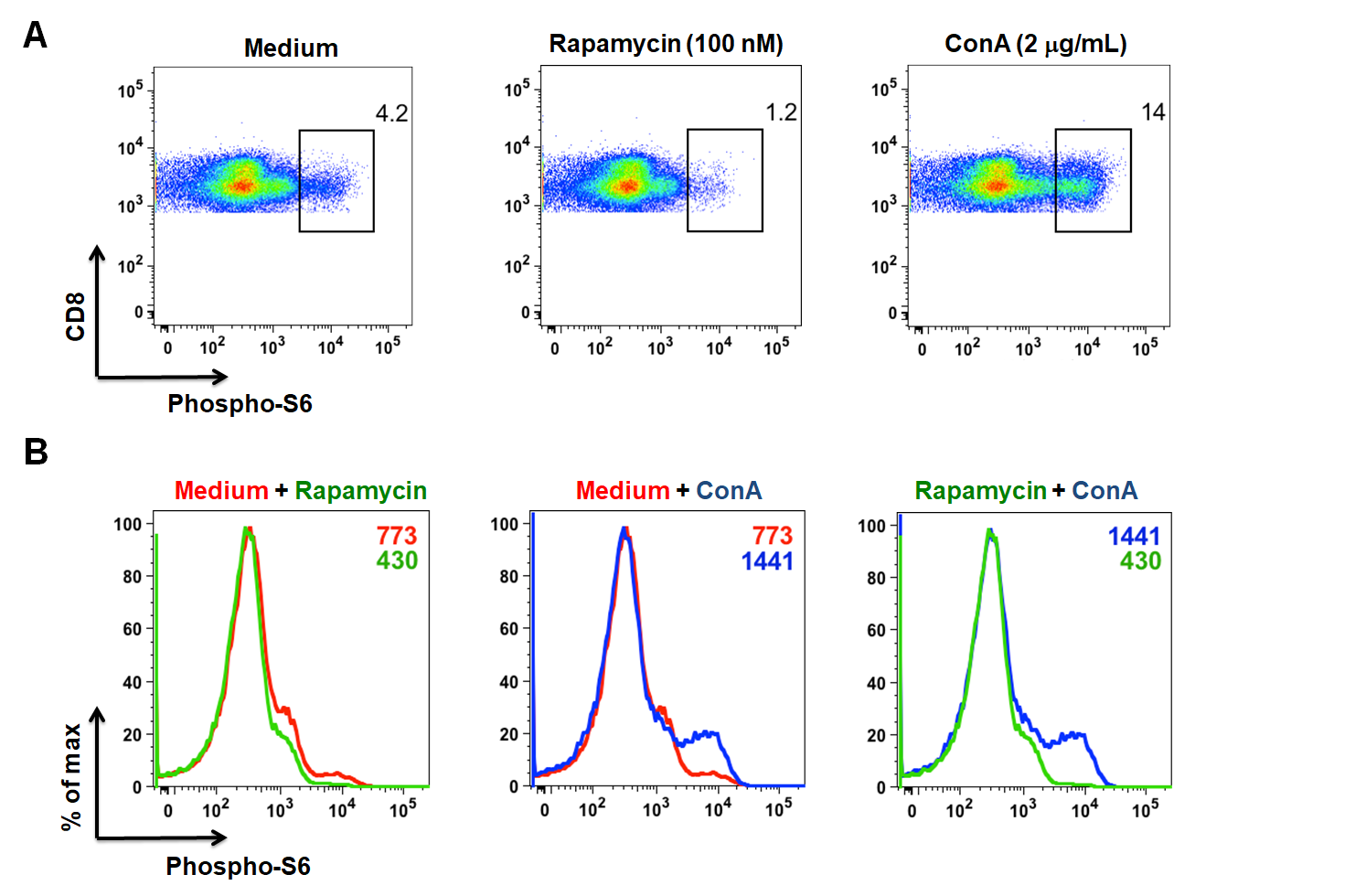

Supplement: Supplementary Figure 1 — Expression of Ribosomal Phospho-S6 (PS6) protein in CD8+ T cells after treatment with rapamycin. Splenocytes from naïve mice were incubated with Concavalin A (2 mg/mL) or rapamycin (100 nM) for one hour. Then, cells were stained with anti-CD8, fixed and permeabilizated to anti-pS6K staining. (A) Dot-plots show the frequency of pS6K in singlets in each condition. (B) Histograms indicate the mean of fluorescence intensity of cells in medium (red line), treated with rapamycin (green line) or stimulated with ConA (blue line). [file Image_1.tif]

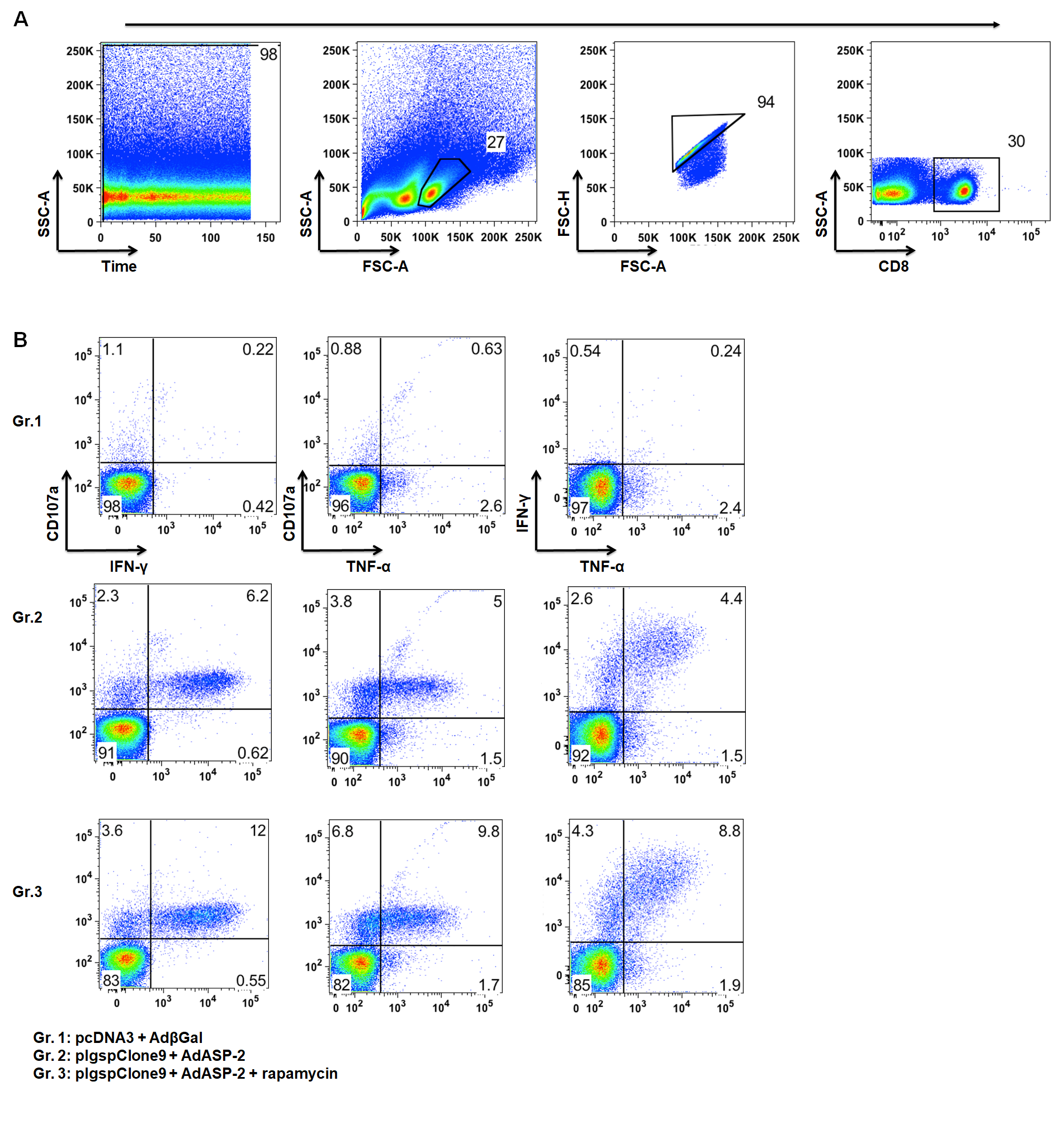

Supplement: Supplementary Figure 2 — Strategy used for analysis of intracellular staining of cytokines in CD8+ T cells. Splenocytes were treated conform described in the methods section. (A) Gate strategy used for positive selection of CD8+ cells. (B) Dot-plots represent the frequencies of CD107a, IFNg and TNF in CD8+ T cells from immunized mice after stimulation with the specific peptide in vitro. Data correspondent to a representative mouse. [file Image_2.tif]

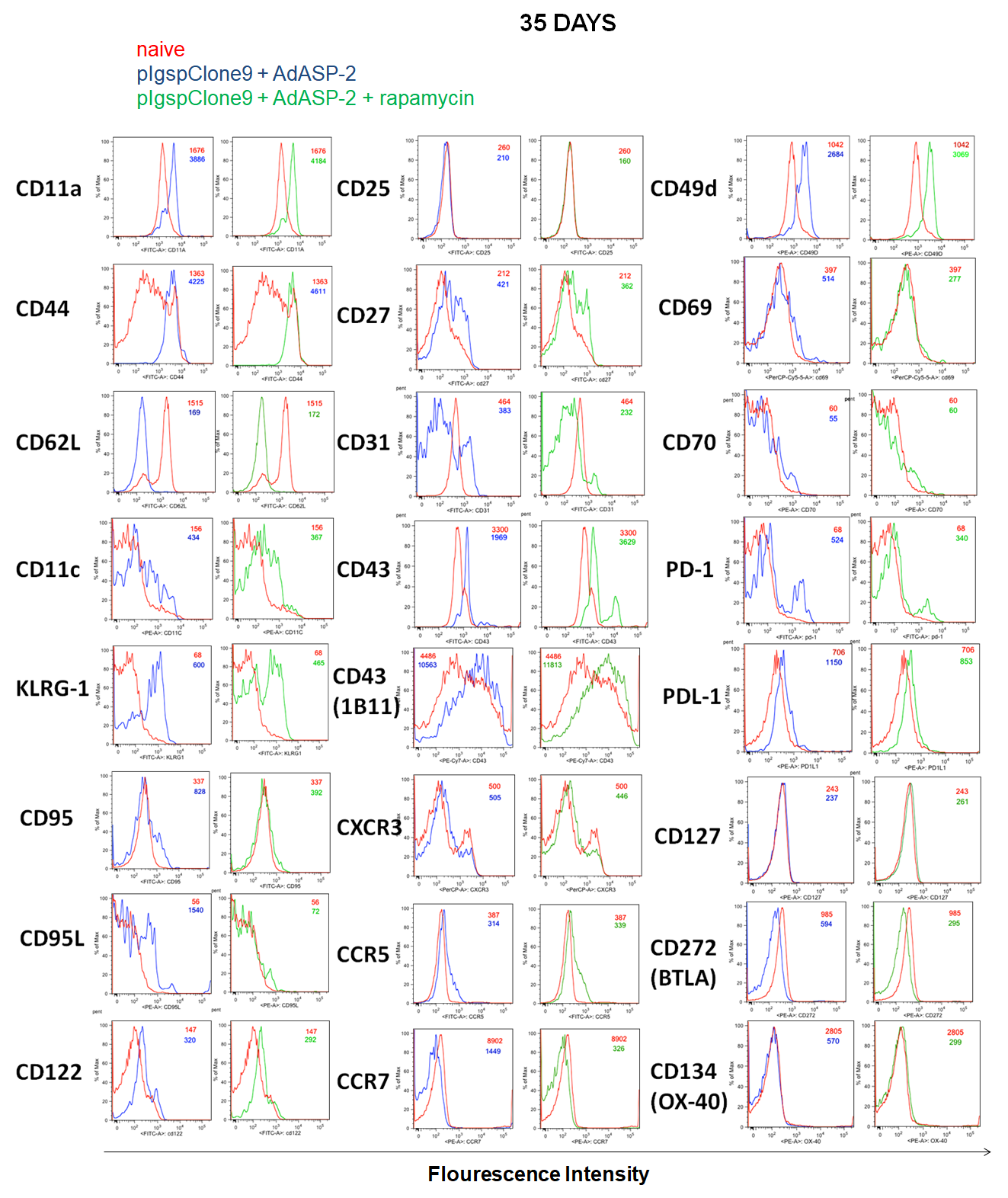

Supplement: Supplementary Figure 3 — Immunophenotyping of specific CD8+ T cells from C57BL/6 mice immunized and treated with rapamycin or diluent after 35 days from priming. C57BL/6 mice were immunized via i.m. with plasmid (100 µg) and adenovirus (2 x 10^8^ pfu) according to the experimental groups described in the method section. They were also treated daily with rapamycin or vehicle (i.p.) for 34 days. After 35 days from priming, splenocytes were labeled with anti-CD8, H2Kb-VNHRFTLV multimer and with the specific markers indicated above for flow cytometric analysis. Histograms show the expression of the markers in CD8+ H2Kb-VNHRFTLV+ cells (blue and green lines) or CD8+ cells of naive as control (red lines). Analyses were performed using cells pools of 4 mice and they are representative of two independent experiments. The numbers indicate the mean fluorescence intensity (MFI). The individual analysis of mice from each group presented similar results. [file Image_3.tif]

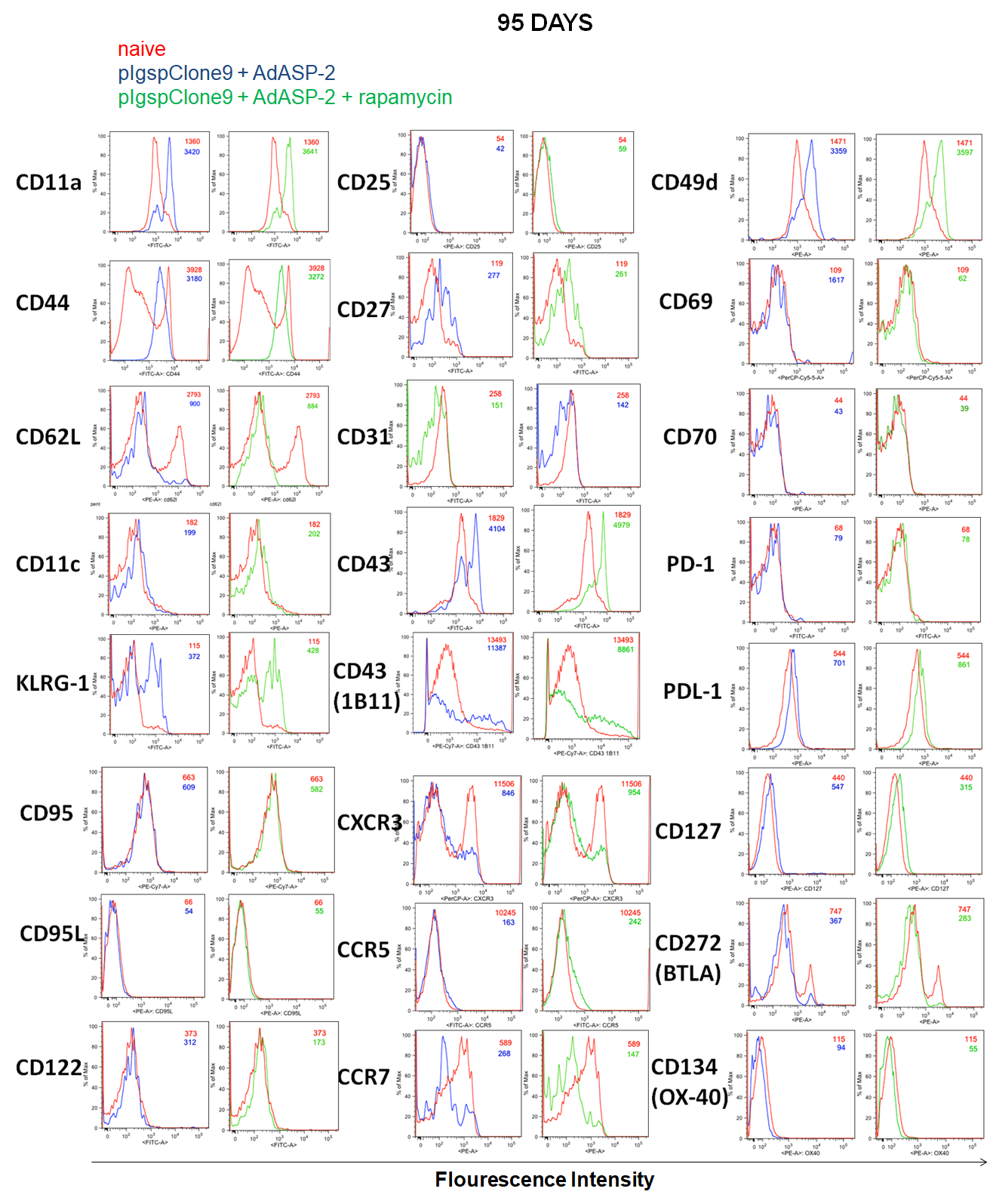

Supplement: Supplementary Figure 4 — Immunophenotyping of specific CD8+ T cells of C57BL/6 mice immunized and treated with rapamycin or diluent after 95 from priming. C57BL/6 mice were immunized via i.m. with plasmid (100 µg) and adenovirus (2 x 10^8^ pfu) according to the experimental groups described in the method section. They were also treated daily with rapamycin or vehicle (i.p.) for 34 days. After 35 days from priming, splenocytes were labeled with anti-CD8, H2Kb-VNHRFTLV multimer and with the specific markers indicated above for flow cytometric analysis. Histograms show the expression of the markers in CD8+ H2Kb-VNHRFTLV+ cells (blue and green lines) or CD8+ cells of naive as control (red lines). Analyses were performed using cells pools of 4 mice and they are representative of two independent experiments. The numbers indicate the mean fluorescence intensity (MFI). The individual analysis of mice from each group presented similar results. [file Image_4.tif]

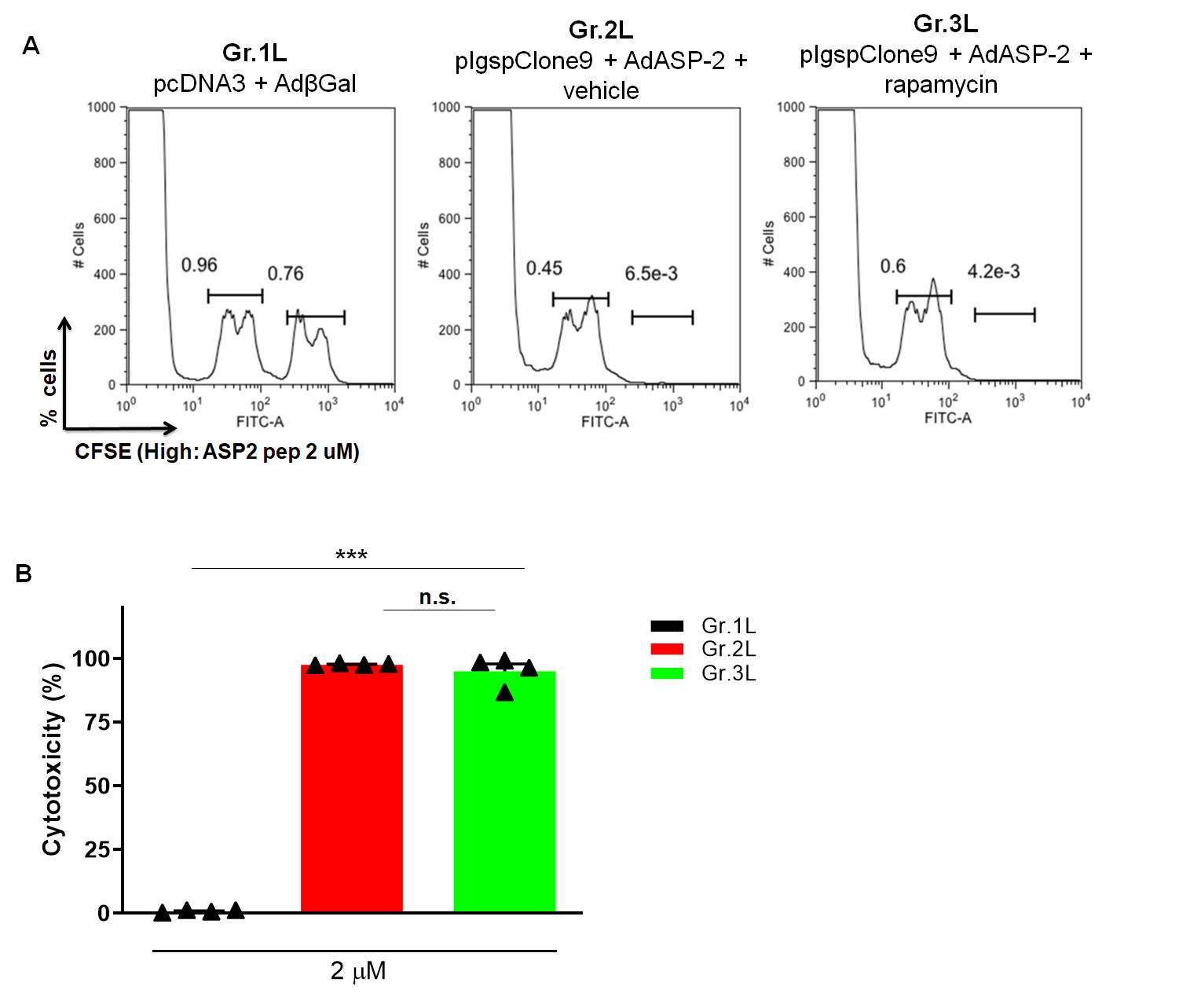

Supplement: Supplementary Figure 5 — In vivo cytotoxicity of specific CD8+ T cells of C57BL/6 mice immunized and treated with rapamycin or diluent. C57BL/6 mice were immunized via i.m. with plasmid (100 µg) and adenovirus (2 x 10^8^ pfu) according to the experimental groups described in the method section. They were also treated daily with rapamycin or vehicle (i.p.) for 34 days. Splenocytes from naive mice were stained with CFSE in 2 different concentrations. CFSEHigh population was pulsed with peptide VNHRFTLV at a final concentration of 2 mM. CFSELow was the negative control. Stained cells were transferred to the experimental groups and, after 14 hours, spleens were harvested to quantify the frequency of stained cells. (A) Histograms represent the frequencies of CFSEHigh and CFSELow in each group. (B) Percentage of CD8+ T mediated cytotoxicity, with mean ± SD. Results from one experiment and 4 mice per group. Statistical analysis was performed using the One-Way ANOVA and Tukey’s HSD tests. Asterisks indicate significant differences among groups, defined as *P < 0.05, **P < 0.01, and ***P < 0.001. N.S., Non-significant. [file Image_5.tif]

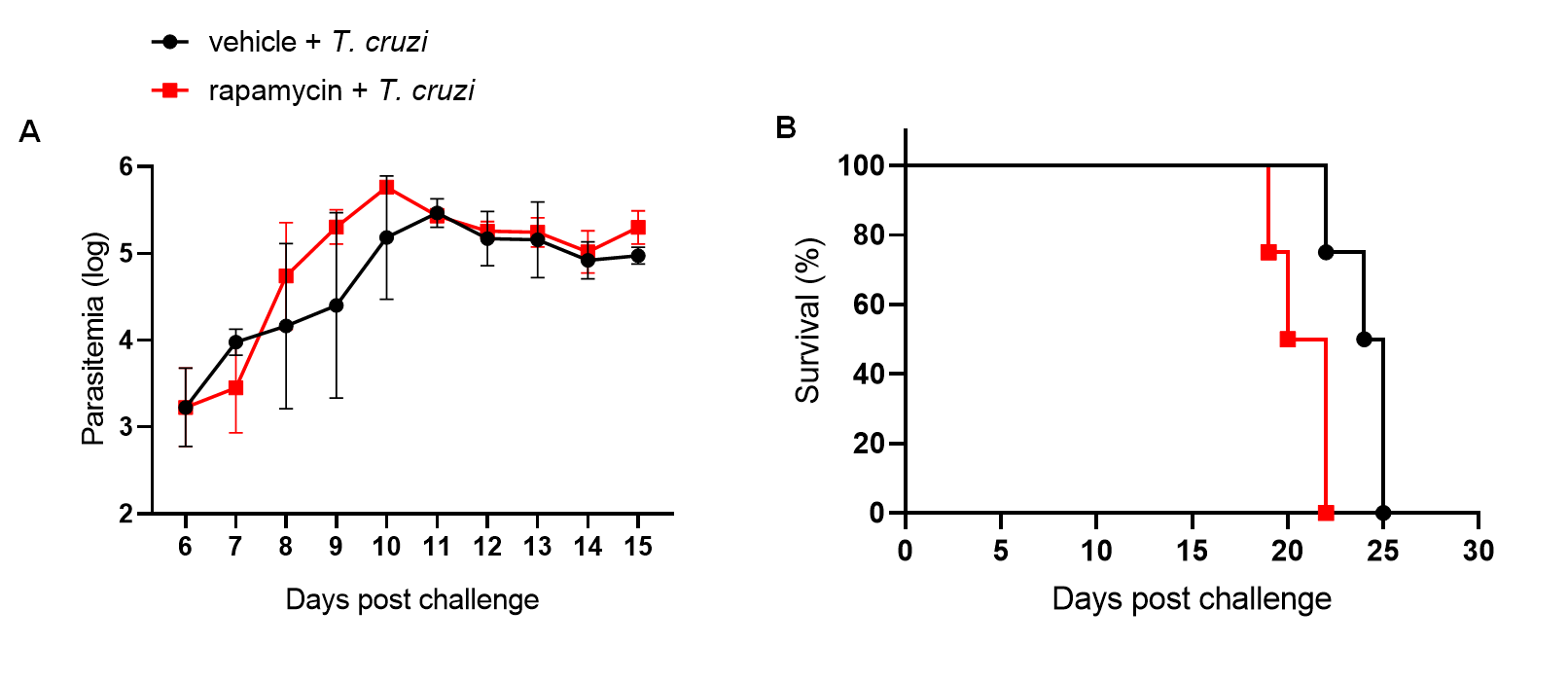

Supplement: Supplementary Figure 6 — Experimental challenge of A/sn mice after treatment with rapamycin or vehicle. A/Sn mice were treated daily with rapamycin or vehicle (PBS) for 34 days. On the last day, mice were infected with 150 blood trypomastigotes of Y strain of T. cruzi. (A) Parasitemia was monitored daily between days 6 and 15 after challenge. The parasitemia values were log transformed. (B) The survival rate was also followed and analyzed by Log-rank (Mantel-Cox) test (all groups p = 0,0285). Results from 4 mice per group. [file Image_6.tif]
